# Supplementary figures and images for: The differentiation of myeloid progenitors is effected by cascading waves of coordinated gene expression that remodel cellular physiology in a characteristic sequence
Source: PLoS Comput Biol. 2026 May 12;22(5):e1014276. doi: 10.1371/journal.pcbi.1014276 (PMC13189416; doi:10.1371/journal.pcbi.1014276)

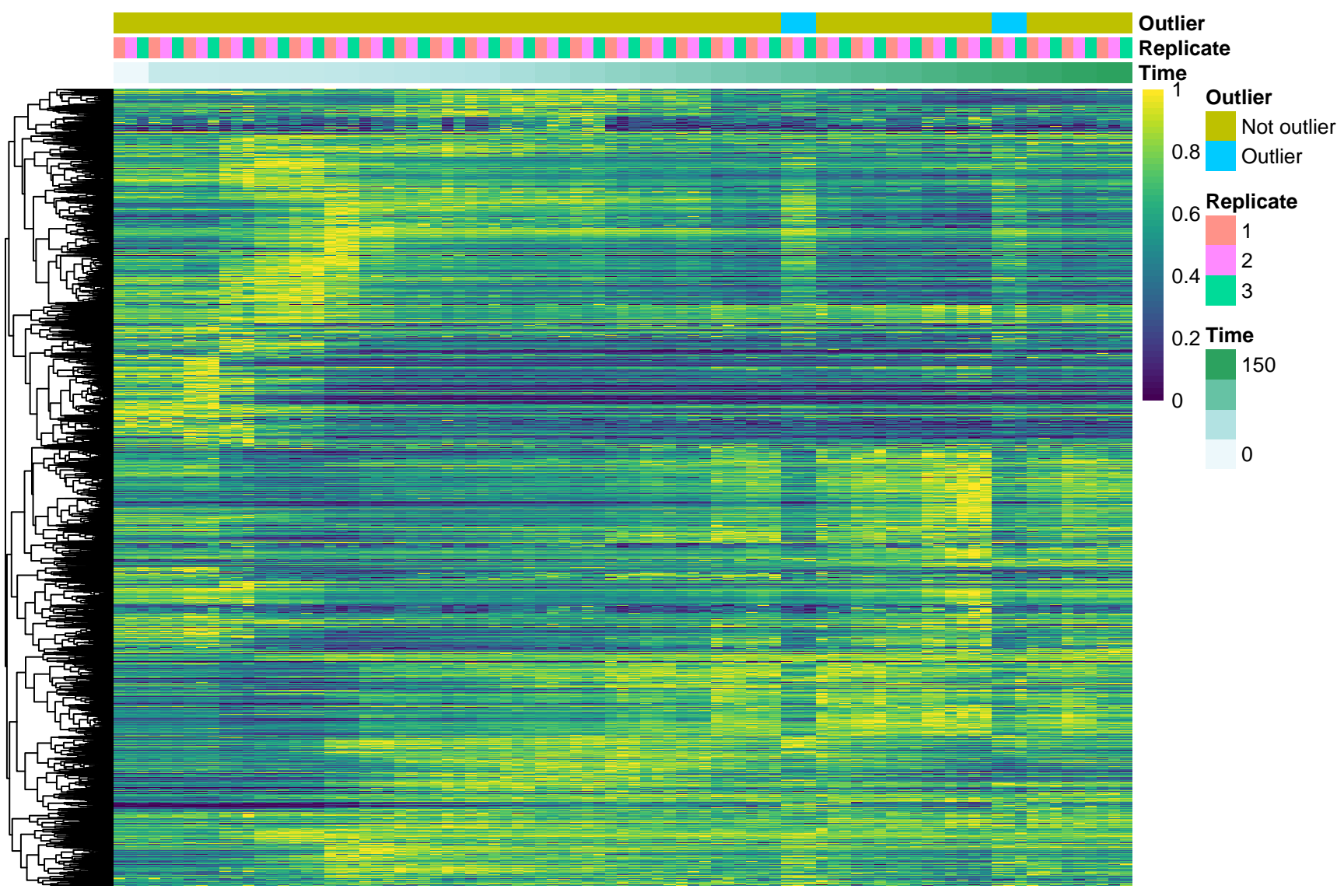

Supplement: S1 Fig — Color indicates the expression of each transcript (y-axis) in each IL3 sample (x-axis) with maximum expression scaled to 1. Transcripts were clustered hierarchically using Pearson correlation as a similarity measure. (PDF) [file pcbi.1014276.s001.pdf]

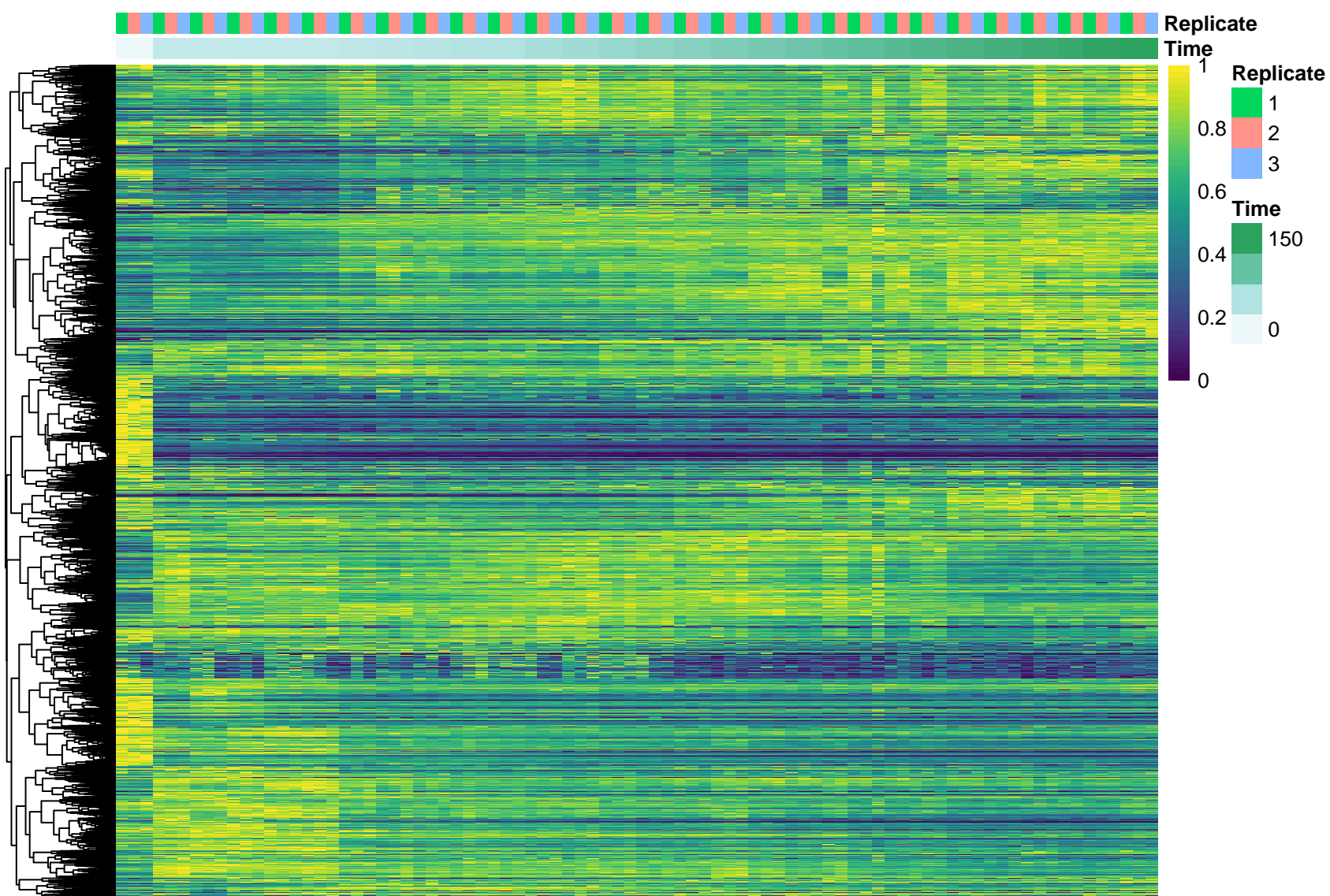

Supplement: S2 Fig — Color indicates the expression of each transcript (y-axis) in each GCSF sample (x-axis) with maximum expression scaled to 1. Transcripts were clustered hierarchically using Pearson correlation as a similarity measure. (PDF) [file pcbi.1014276.s002.pdf]

log2(expression)

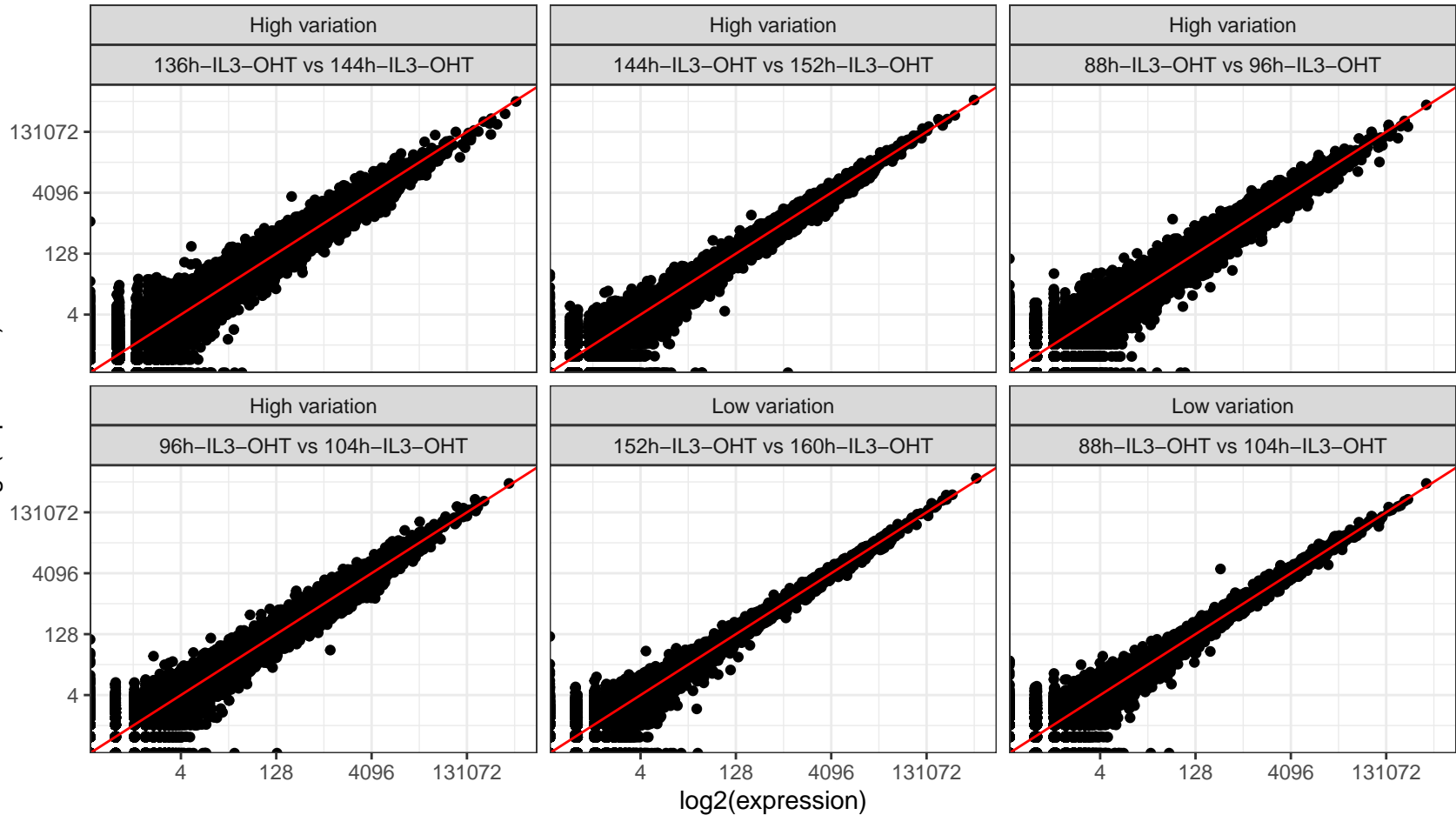

Supplement: S3 Fig — Correlation in gene expression between suspected outliers and immediate neighbors (88h vs 96h, 96h vs 104h, 136h vs 144h, and 144h vs 152h). Correlation between the timepoint immediately preceding and the timepoint immediately following the suspected outlier (88h vs 104h and 136h vs 152h). (PDF) [file pcbi.1014276.s003.pdf]

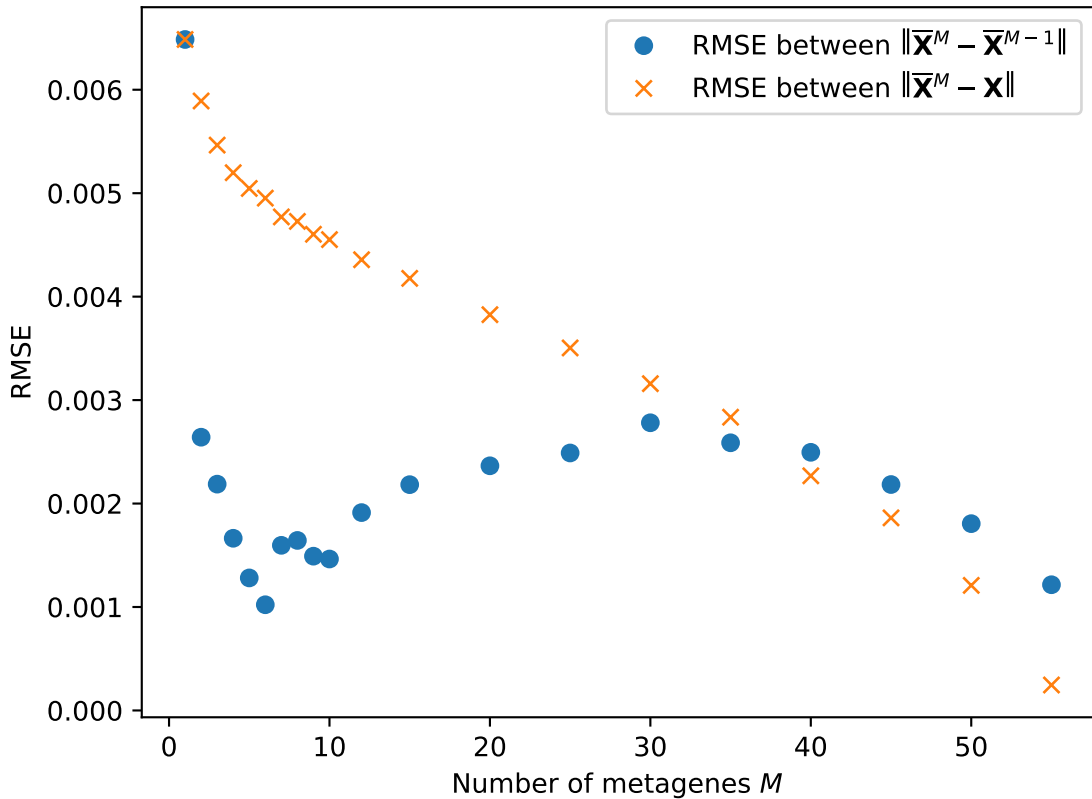

Supplement: S4 Fig — The root mean square error (RMSE) between the gene expression matrix and its NMF approximation (orange crosses) or the RMSE between successive approximations (blue dots) as the number of metagenes (M) is varied. (PDF) [file pcbi.1014276.s004.pdf]

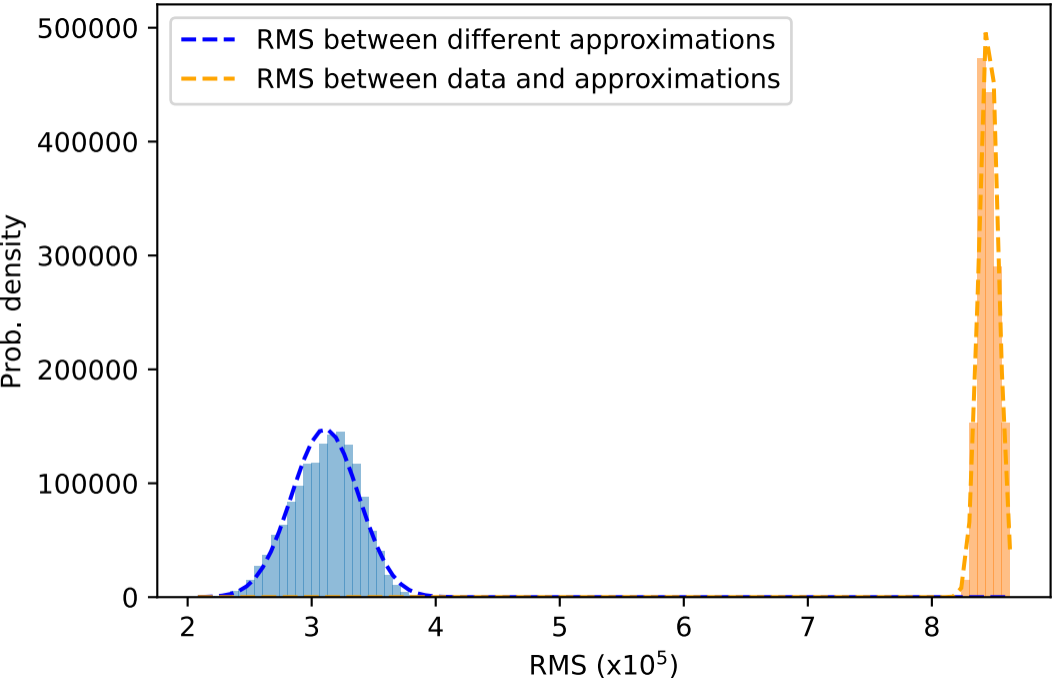

Supplement: S5 Fig — Histograms of the RMSE between the gene expression matrix and 100 NMF approximations (orange) or the RMSE between the approximations (blue). Dashed lines are the Gaussian densities with the mean and standard deviation of each histogram. (PDF) [file pcbi.1014276.s005.pdf]

**A**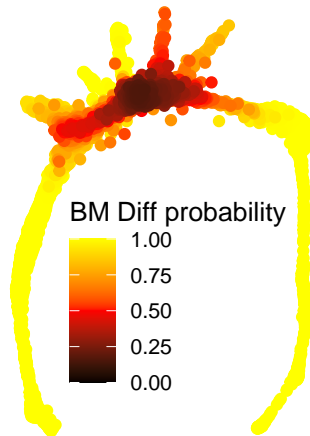**B**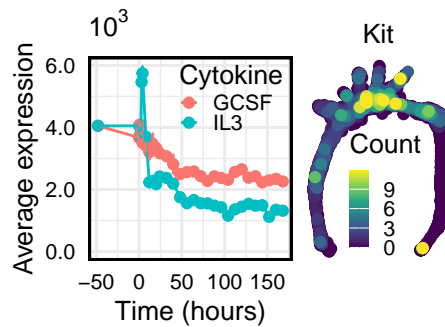**C**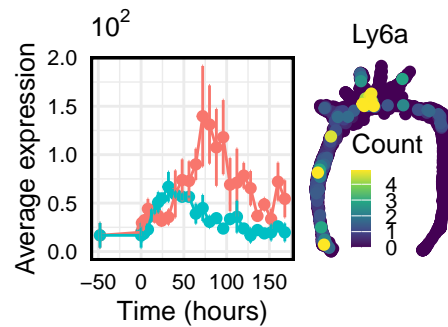**D**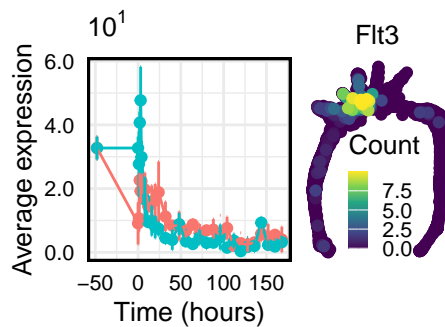

Supplement: S6 Fig — A. SPRING plot of BM cells. Cells are colored according to the probability of differentiation computed by Tusi et al. [16], which is inversely related to the probability of a cell being an HSC. B-D. Gene expression of HSC markers during PUER differentiation (left) and in BM (right; SPRING plot). Errors bars indicate standard deviation. (PDF) [file pcbi.1014276.s006.pdf]

**A**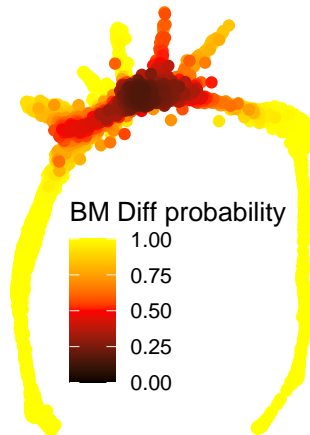**B**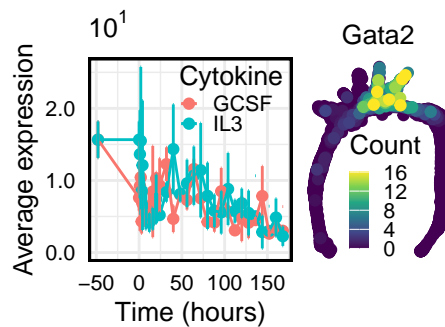**C**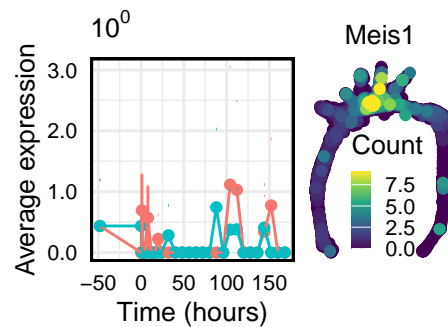**D**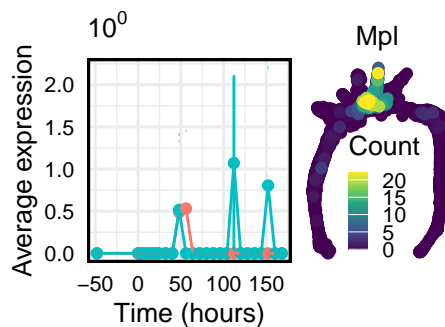**E**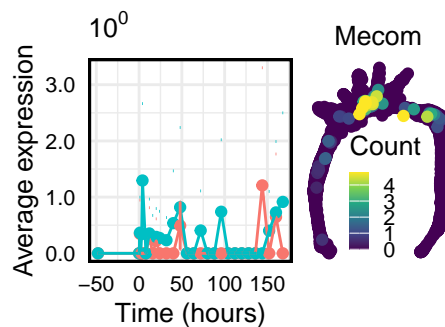**F**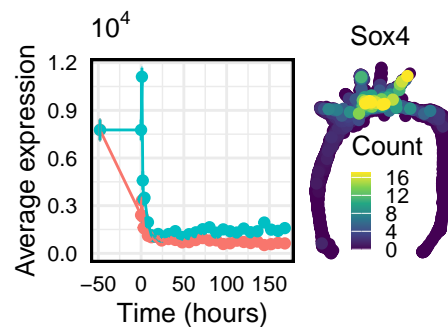

Supplement: S7 Fig — A. SPRING plot of BM cells. Cells are colored according to the probability of differentiation computed by Tusi et al. [16], which is inversely related to the probability of a cell being an HSC. B-F. Gene expression of HSC TFs during PUER differentiation (left) and in BM (right; SPRING plot). Errors bars indicate standard deviation. (PDF) [file pcbi.1014276.s007.pdf]

**A**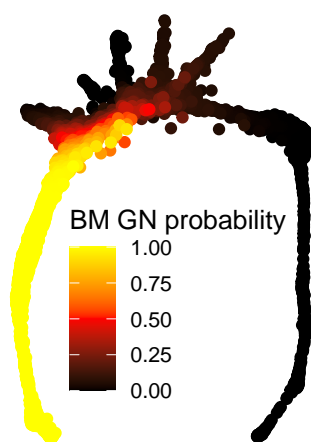**B**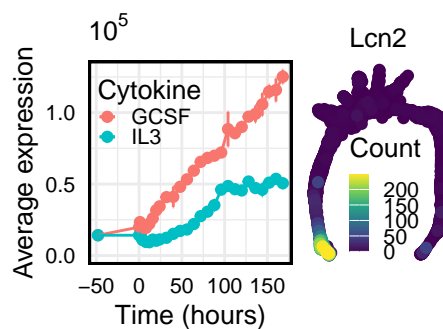**C**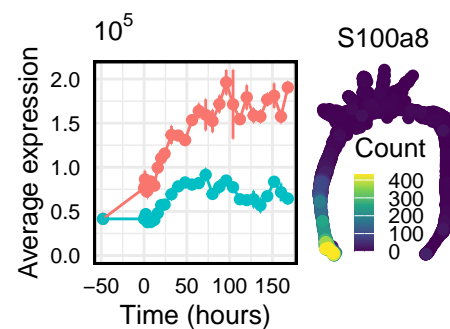**D**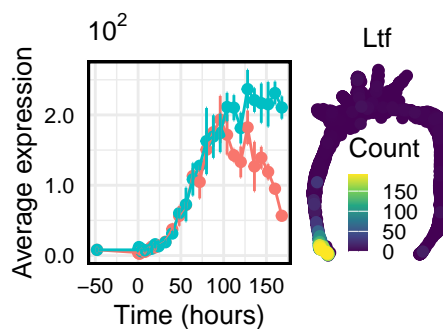**E**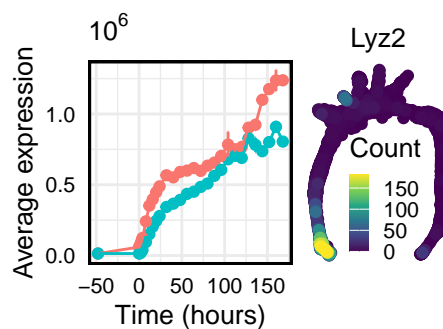**F**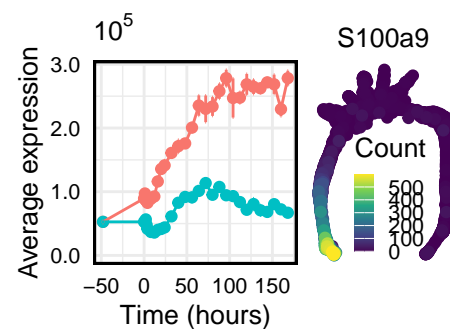**G**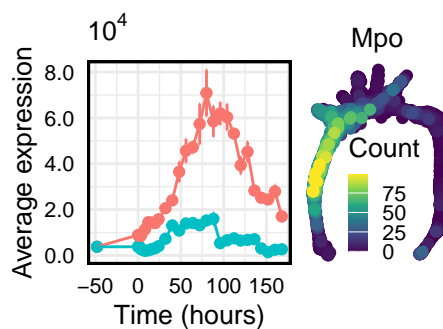**H**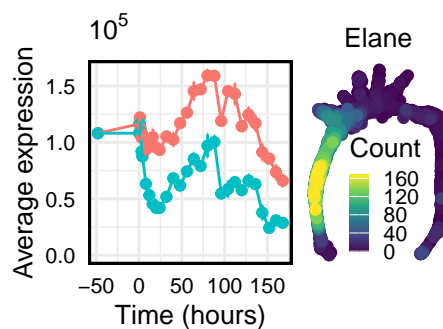**I**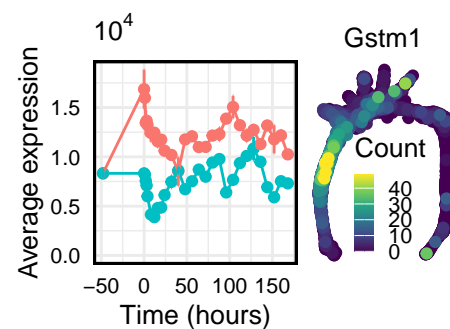**J**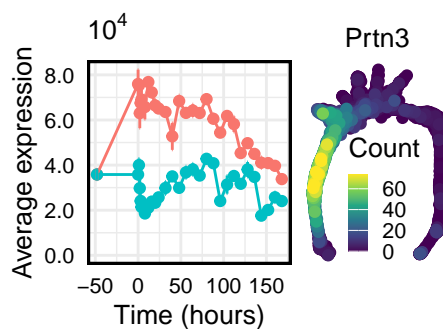**K**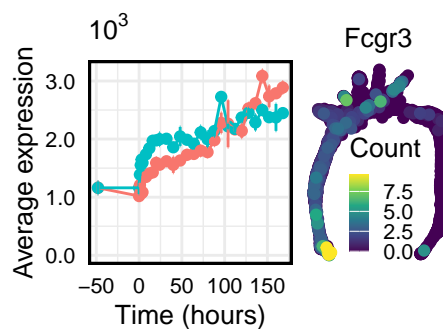**L**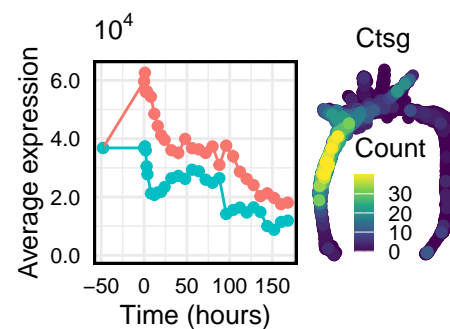

Supplement: S8 Fig — A. SPRING plot of BM cells. Cells are colored according to the PBA probability of being a neutrophil. B-L. Gene expression of neutrophils markers during PUER differentiation (left) and in BM (right; SPRING plot). Errors bars indicate standard deviation. (PDF) [file pcbi.1014276.s008.pdf]

**A**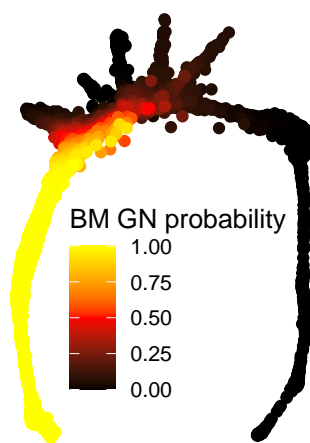**B**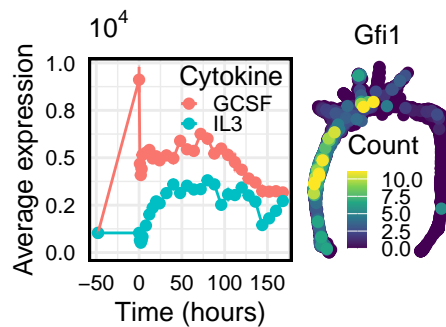**C**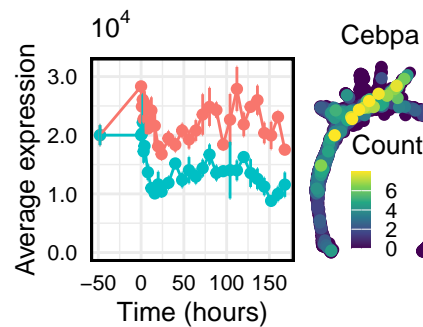**D**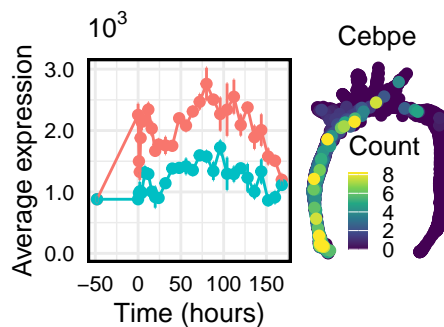**E**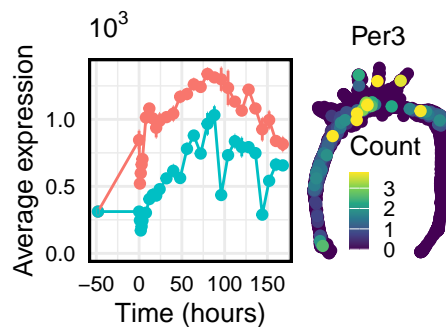

Supplement: S9 Fig — A. SPRING plot of BM cells. Cells are colored according to the PBA probability of being a neutrophil. B-E. Gene expression of neutrophil TFs during PUER differentiation (left) and in BM (right; SPRING plot). Errors bars indicate standard deviation. (PDF) [file pcbi.1014276.s009.pdf]

**A**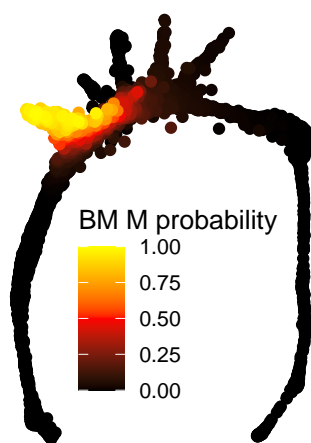**B**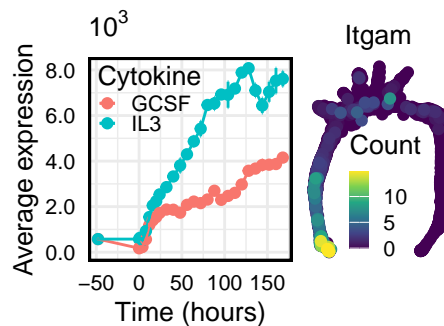**C**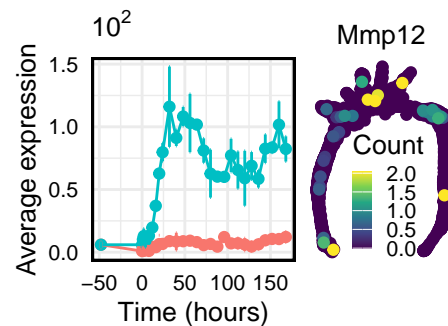**D**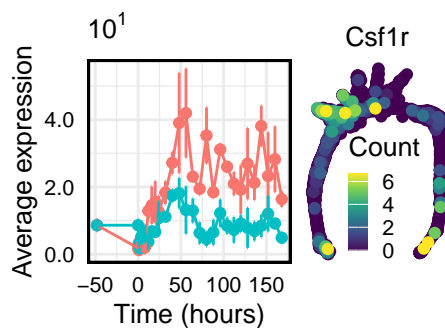**E**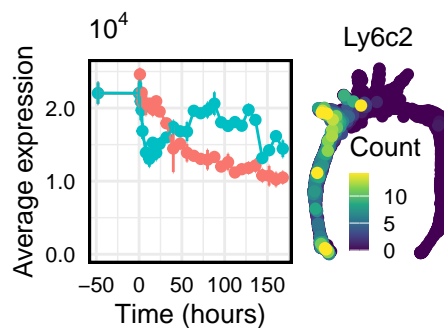**F**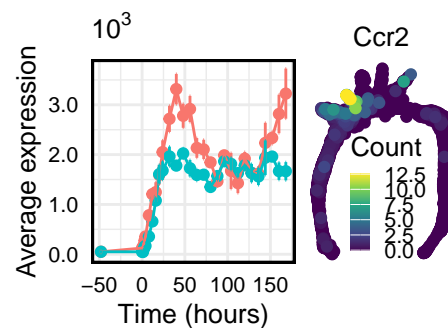**G**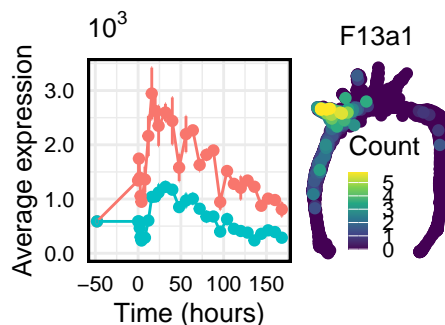**H**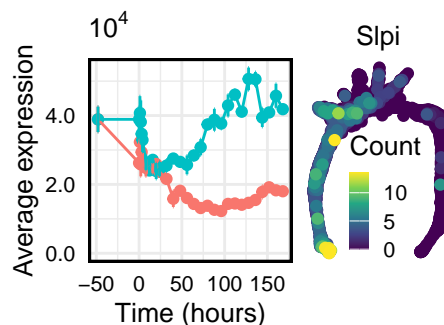**I**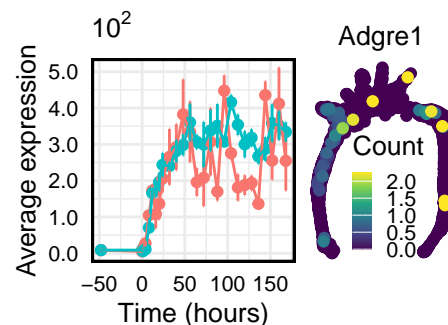

Supplement: S10 Fig — A. SPRING plot of BM cells. Cells are colored according to the PBA probability of being a macrophage. B-I. Gene expression of macrophage markers during PUER differentiation (left) and in BM (right; SPRING plot). Errors bars indicate standard deviation. (PDF) [file pcbi.1014276.s010.pdf]

**A**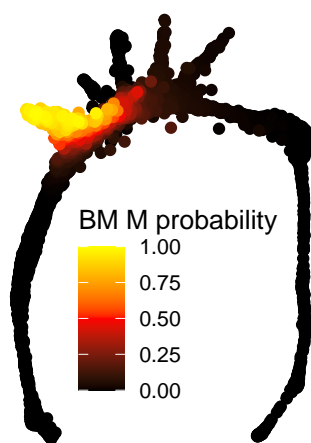**B**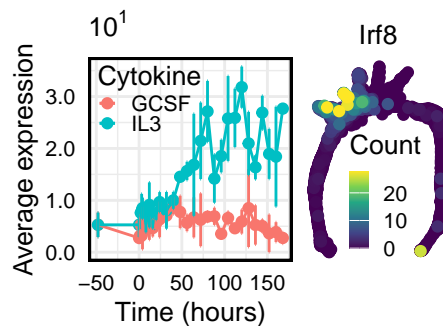**C**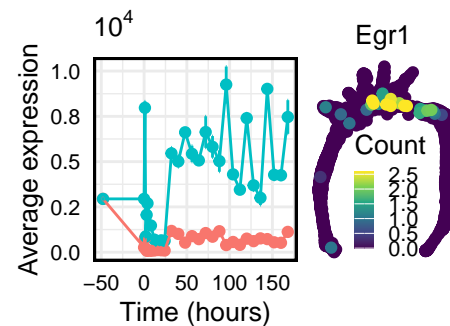**D**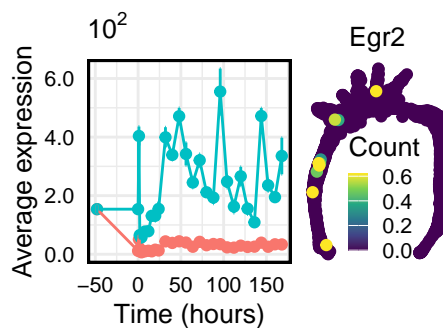**E**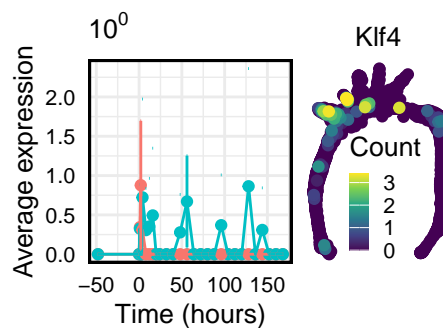**F**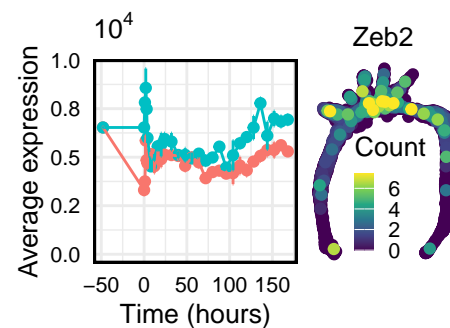**G**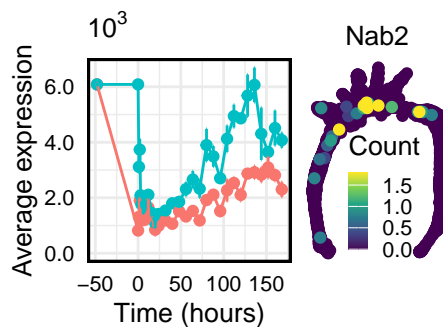

Supplement: S11 Fig — A. SPRING plot of BM cells. Cells are colored according to the PBA probability of being a macrophage. B-G. Gene expression of macrophage TFs during PUER differentiation (left) and in BM (right; SPRING plot). Errors bars indicate standard deviation. (PDF) [file pcbi.1014276.s011.pdf]

**A**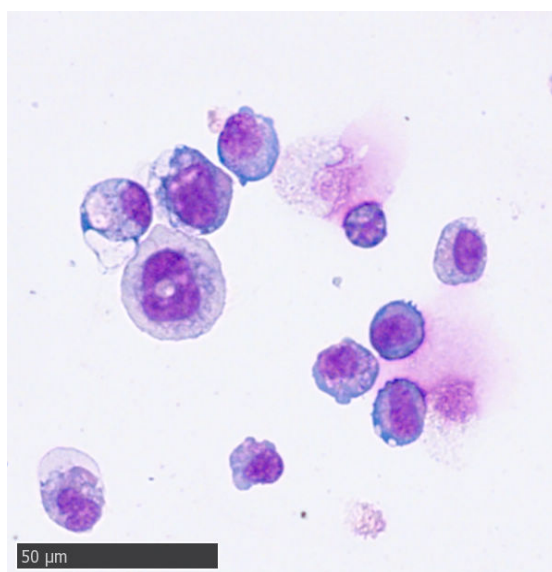**B**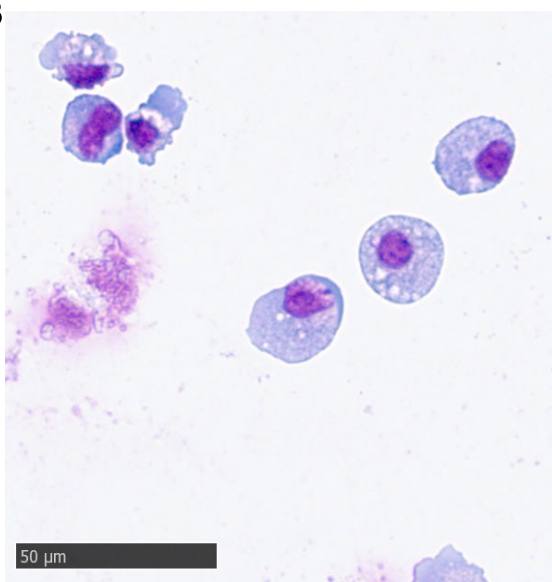**C**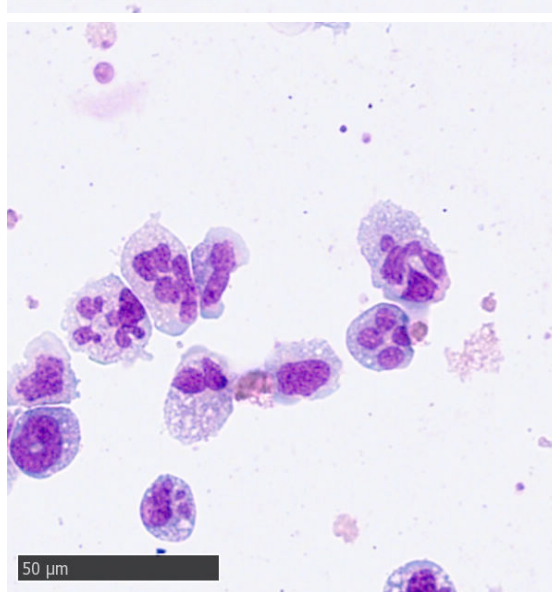**D**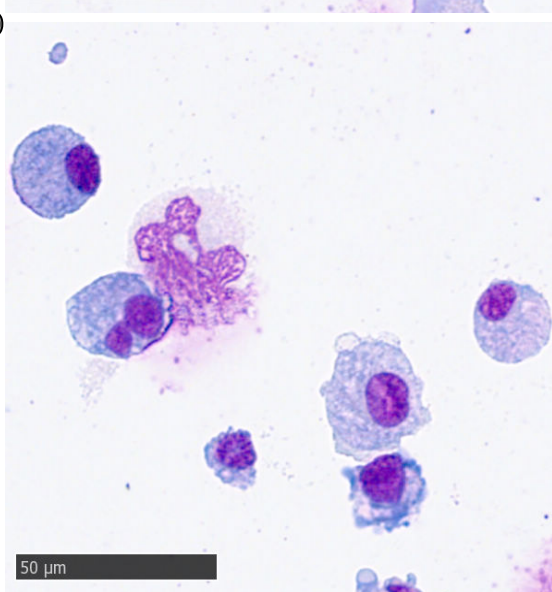**E**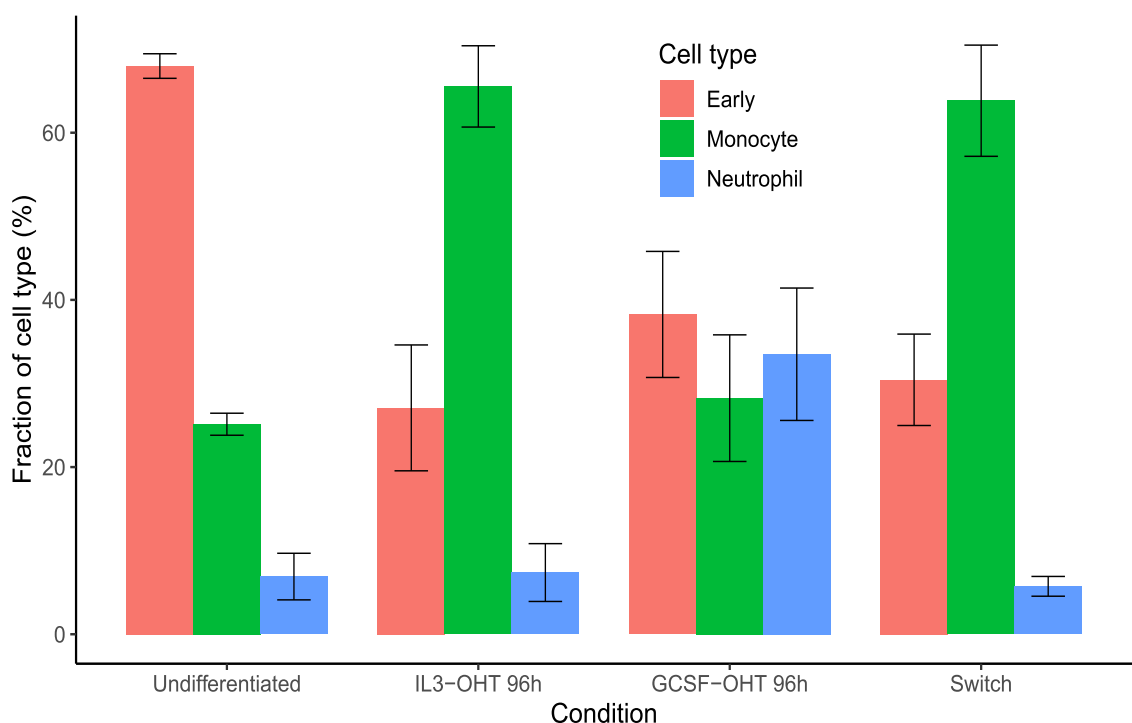

Supplement: S12 Fig — A-D. PUER cells stained with Wright Giemsa. A. Undifferentiated cells B. 96h IL3-OHT (macrophage). C. 96h GCSF-OHT (neutrophil). D. “Switch”. Cells pre-treated with GCSF for 48 hours and then switched to IL3 prior to OHT addition. Cells were treated with OHT for 96 hours. E. The distribution of cell morphology in each treatment. Early or undifferentiated cell types include myeloblasts, monoblasts, and promyelocytes [14,47]. Neutrophils include myelocytes, metamyelocytes, band cells, and segmented neutrophils. Monocytes include both mature monocytes and macrophages. Error bars are ±σ, where σ is the standard deviation computed over two (undifferentiated, switch) or three (96h IL3-OHT, 96h GCSF-OHT) replicates. (PDF) [file pcbi.1014276.s012.pdf]

Undifferentiated

IL3-OHT 96h

GCSF-OHT 96h

Switch

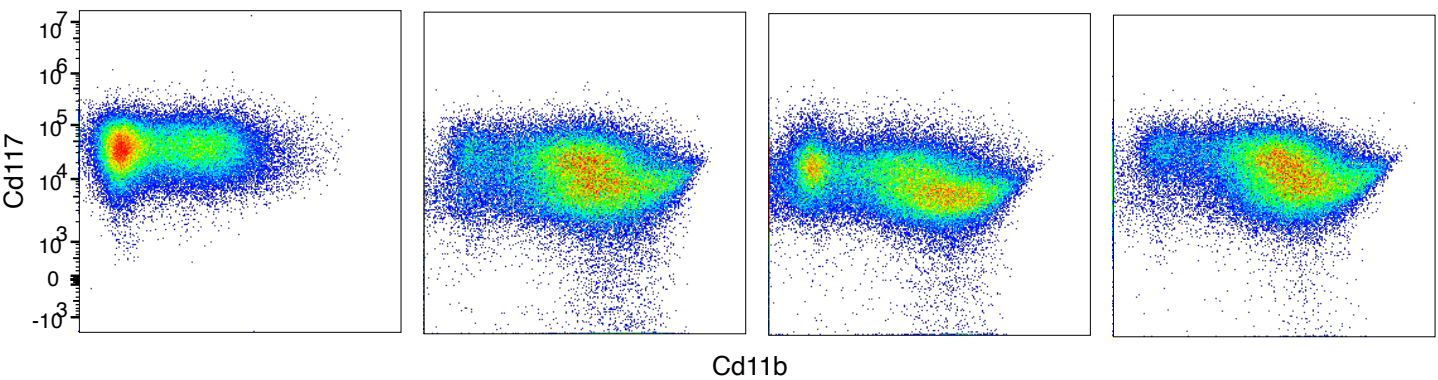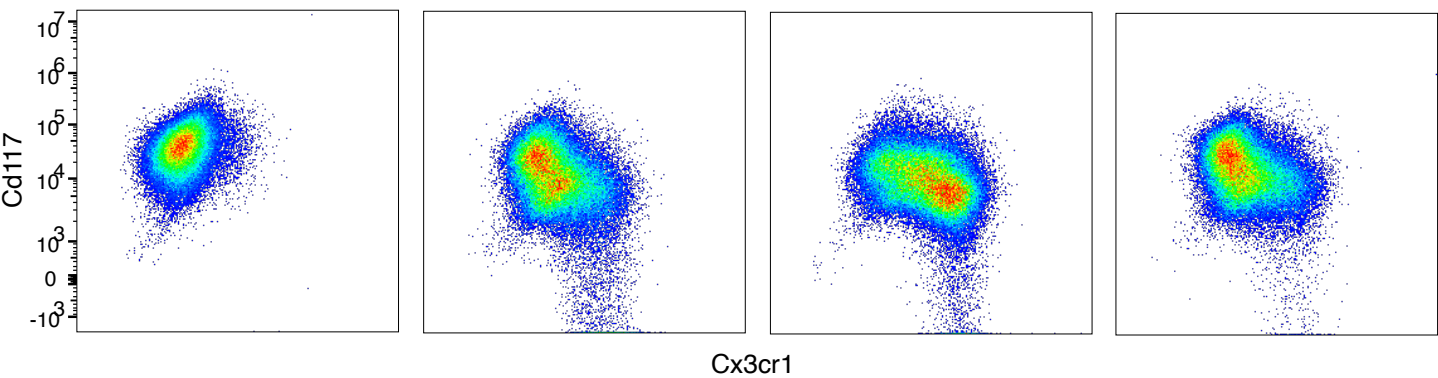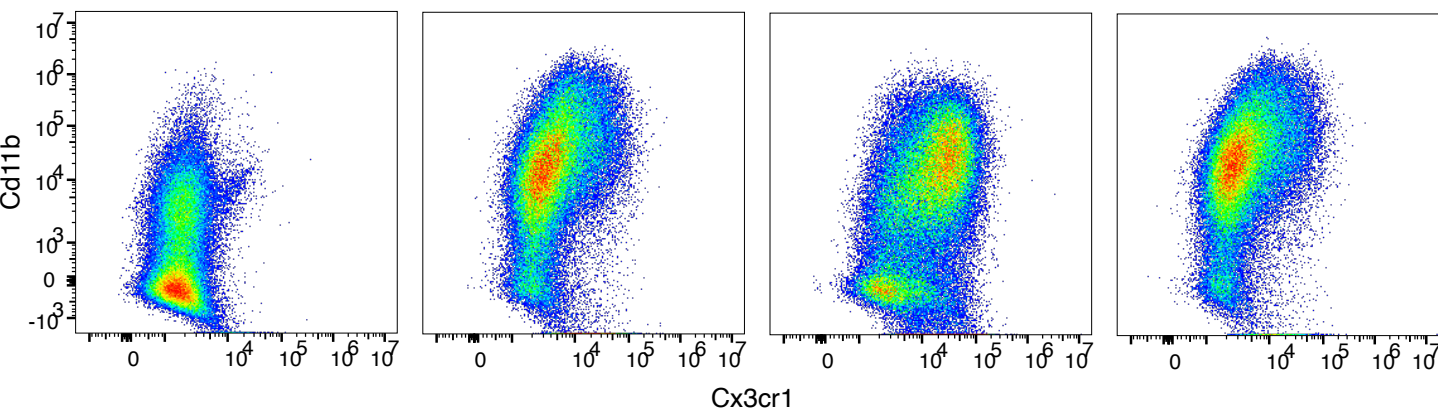

Supplement: S13 Fig — Dot plots of Cd117, Cd11b, and Cx3cr1 expression in Undifferentiated, 96h IL3-OHT (macrophage), 96h GCSF-OHT (neutrophil) and “switch” samples. In the switch experiment, cells were pre-treated with GCSF for 48 hours and then switched to IL3 prior to OHT addition. Cells were treated with OHT for 96 hours. (PDF) [file pcbi.1014276.s013.pdf]

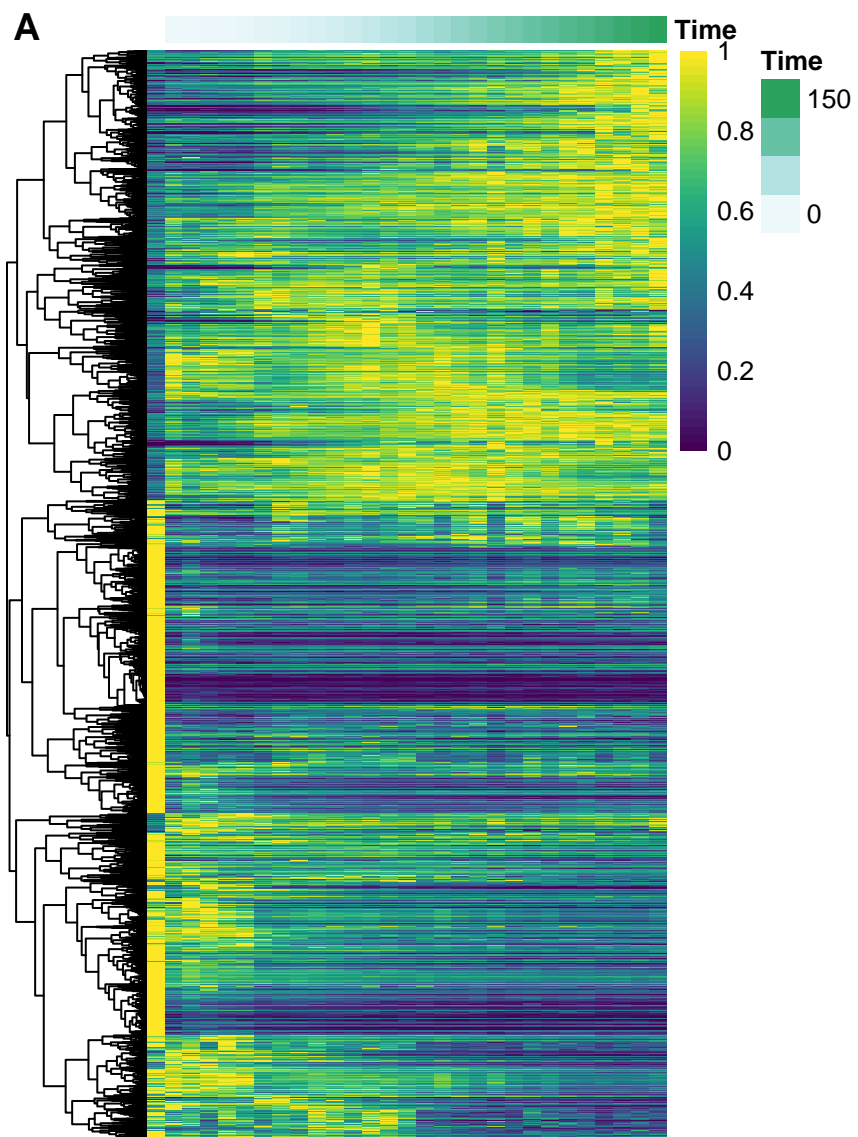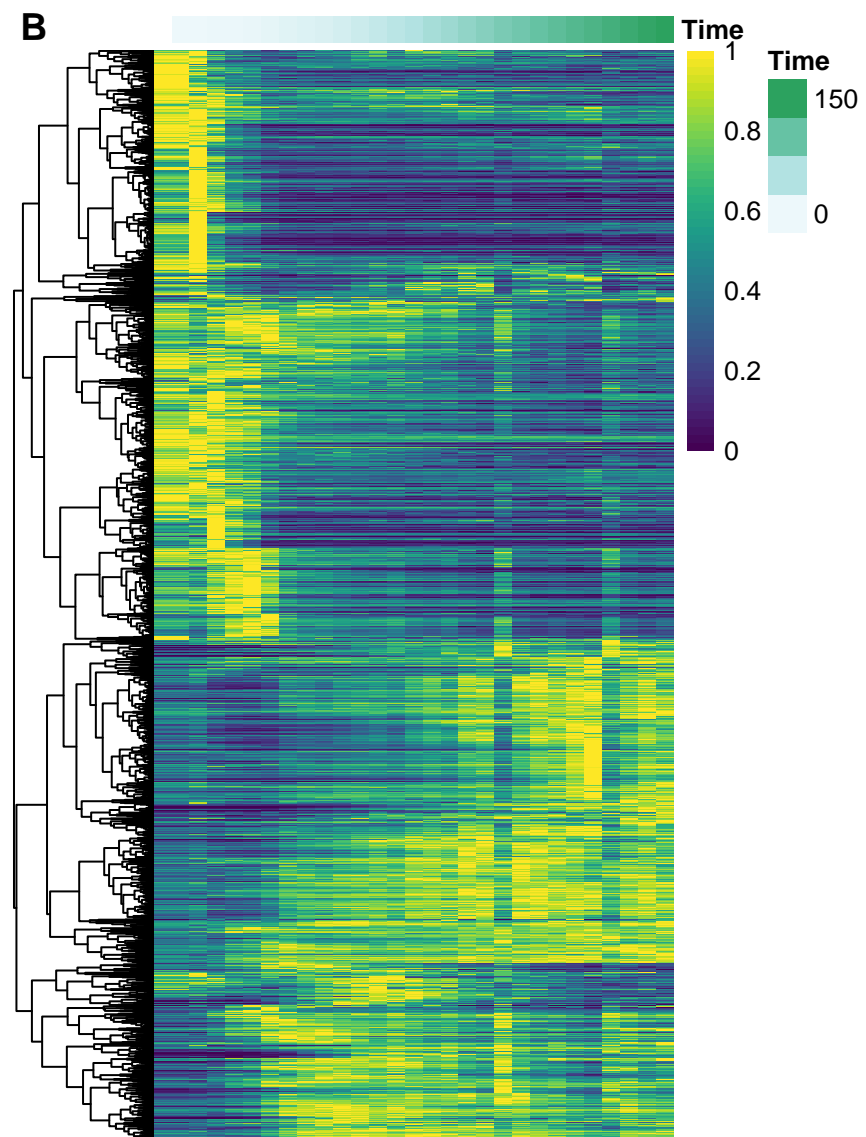

Supplement: S14 Fig — The expression of all differentially expressed genes, with maximum expression scaled to 1, is shown as a color map. Genes are clustered hierarchically according to the Pearson correlation of scaled temporal expression patterns. (A) GCSF. Plotted genes are the union of DEGs identified in the comparisons between the −48h and 168h, −48h and 0h, and 0h and 168h GCSF samples (Fig 3A–C). (B) IL3. DEGs identified in the comparison between the 0h and 168h IL3 samples (Fig 3D). (PDF) [file pcbi.1014276.s014.pdf]

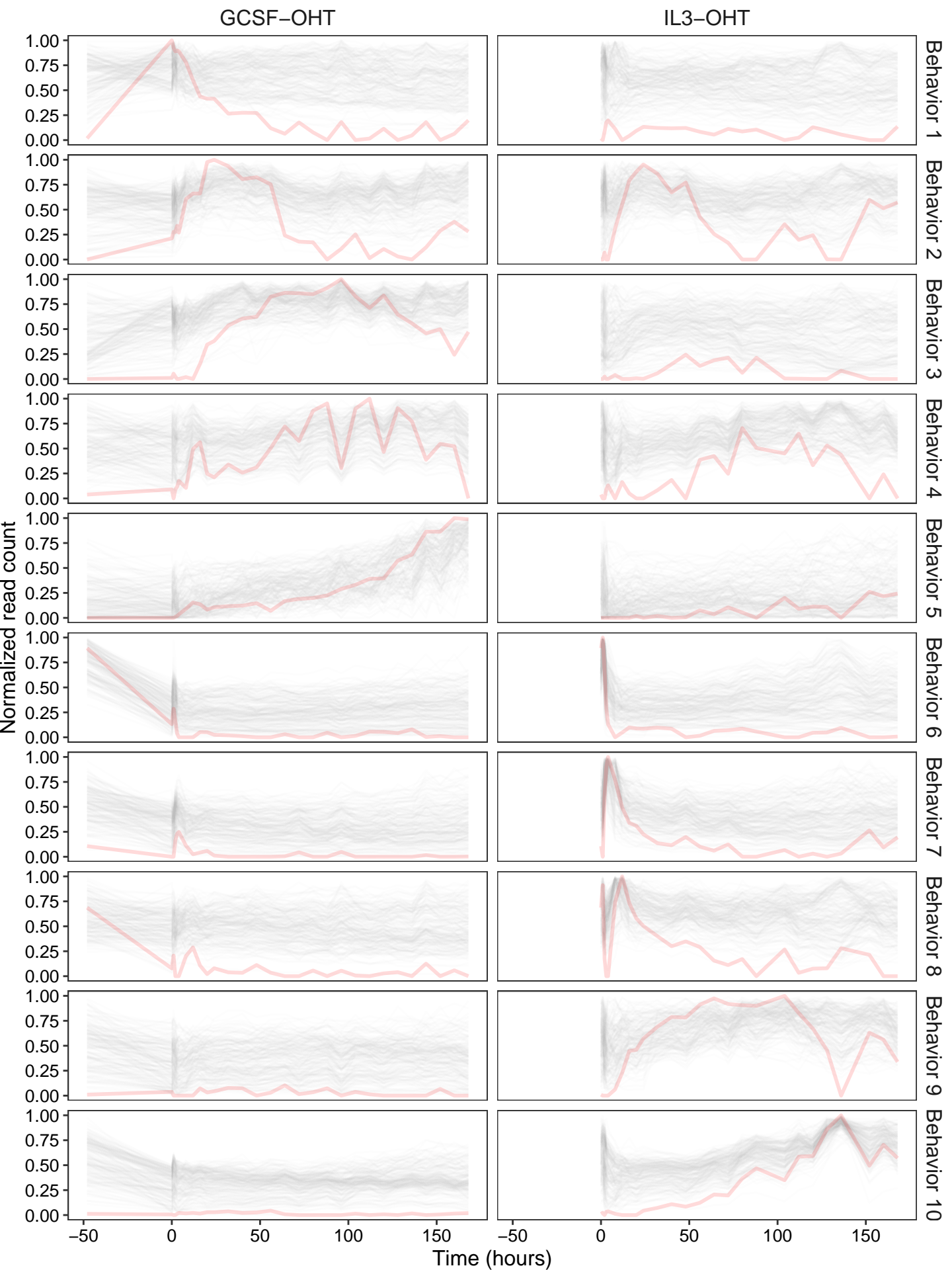

Supplement: S15 Fig — The temporal behavior of each metagene is shown in red. The temporal expression patterns of the transcripts having the highest 200 weights for each behavior are shown in black. (PDF) [file pcbi.1014276.s015.pdf]

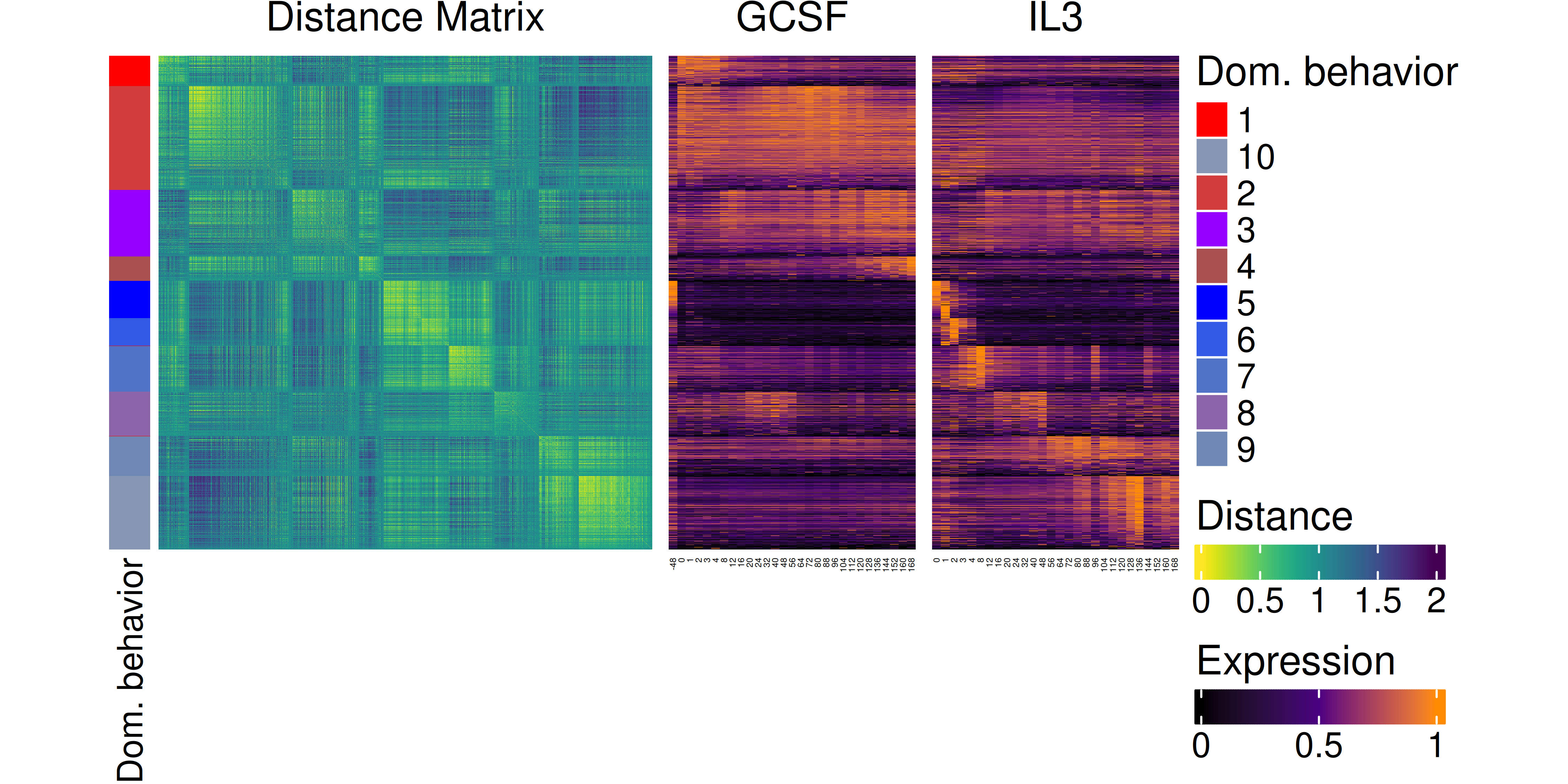

Supplement: S16 Fig — The similarity between the temporal expression of each pair of genes, as measured by the distance 1 − r, where r is Pearson’s correlation coefficient is plotted as a heat map (“distance matrix”). The genes are ordered by their dominant behavior, annotated on the left. The center and right heatmaps show the temporal expression pattern of each genes. (TIFF) [file pcbi.1014276.s016.tiff]

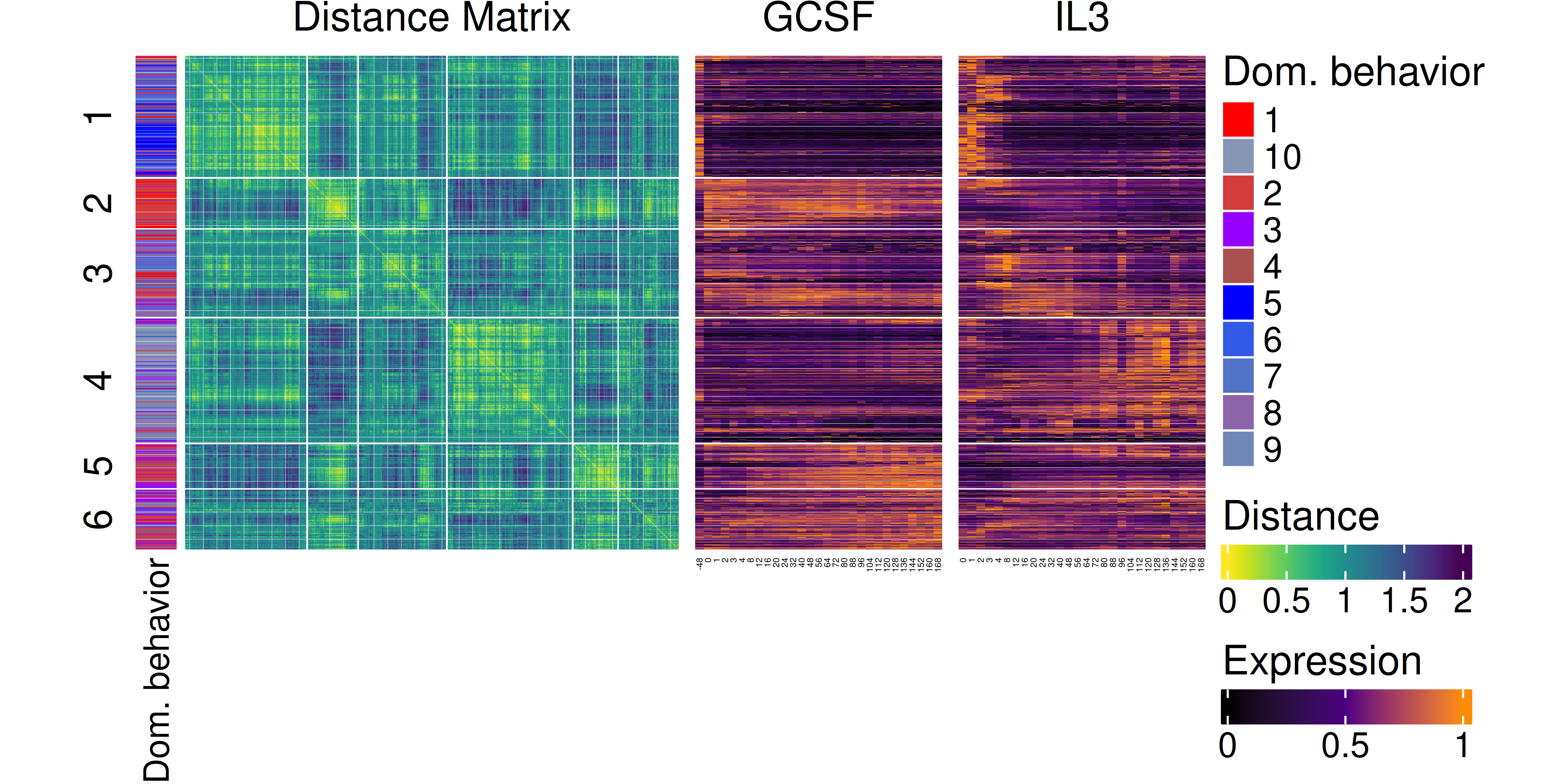

Supplement: S17 Fig — Genes are hierarchically clustered. See the legend of S16 Fig for the plot description. (TIFF) [file pcbi.1014276.s017.tiff]

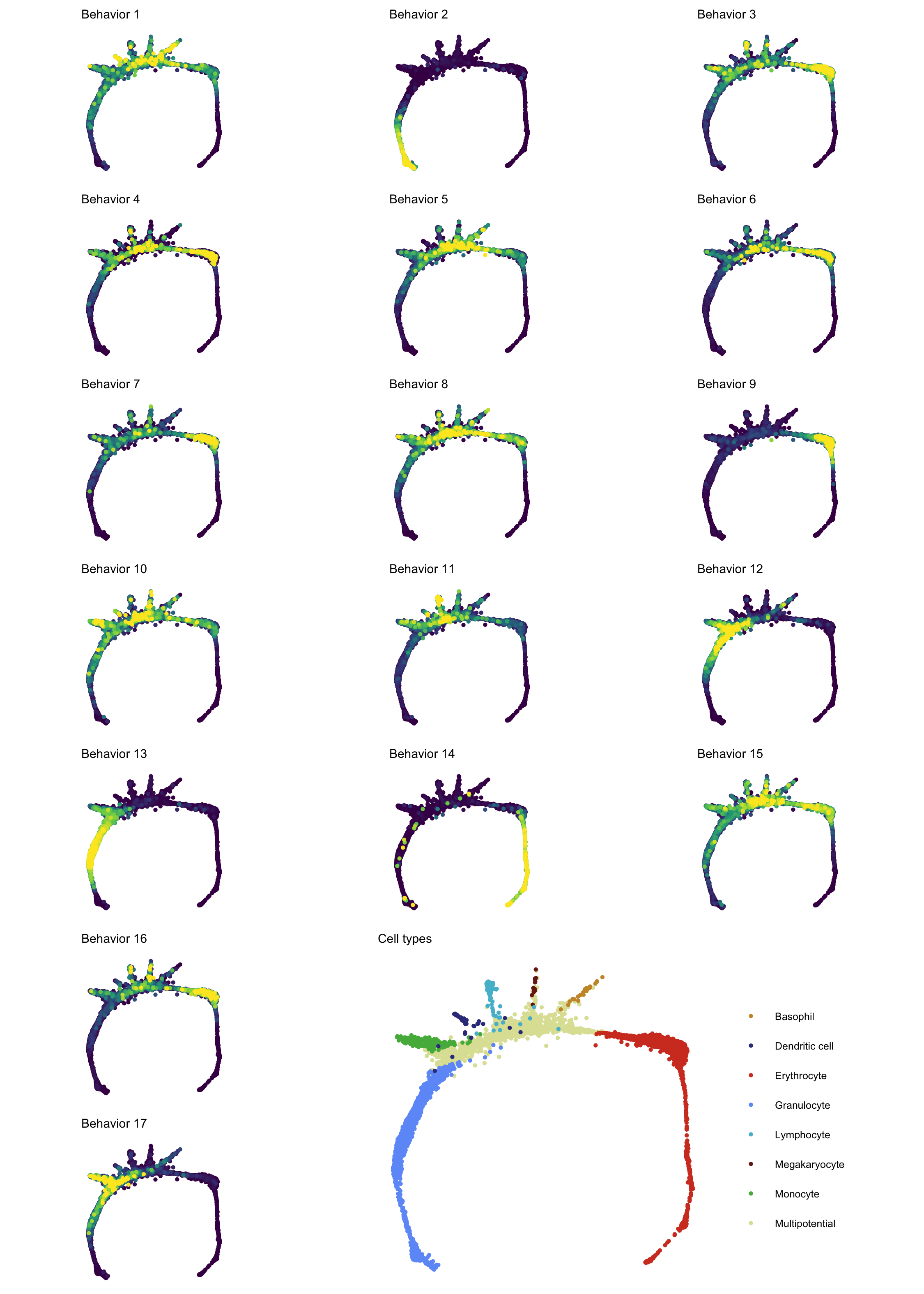

Supplement: S18 Fig — BM progenitor single-cell gene expression data. The behavior in each cell is shown as a SPRING plot. The SPRING plot at the bottom right indicates which cells have greater than 80% PBA probability of belonging to each lineage. Gene expression was scaled so that the maximum for each gene is 1. (TIFF) [file pcbi.1014276.s018.tiff]

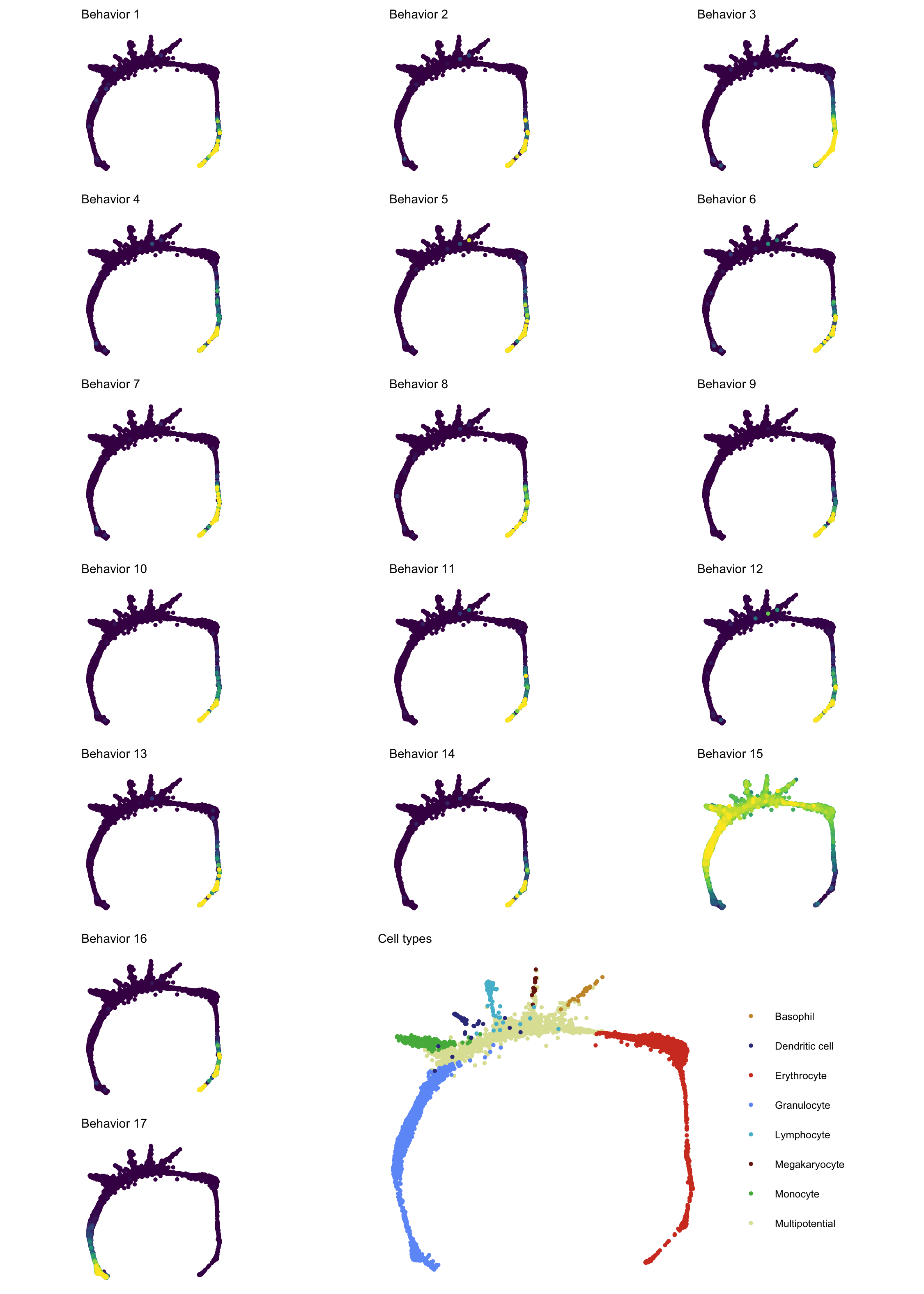

Supplement: S19 Fig — BM progenitor single-cell gene expression data without prior scaling. See the legend of S18 Fig for description. (TIFF) [file pcbi.1014276.s019.tiff]

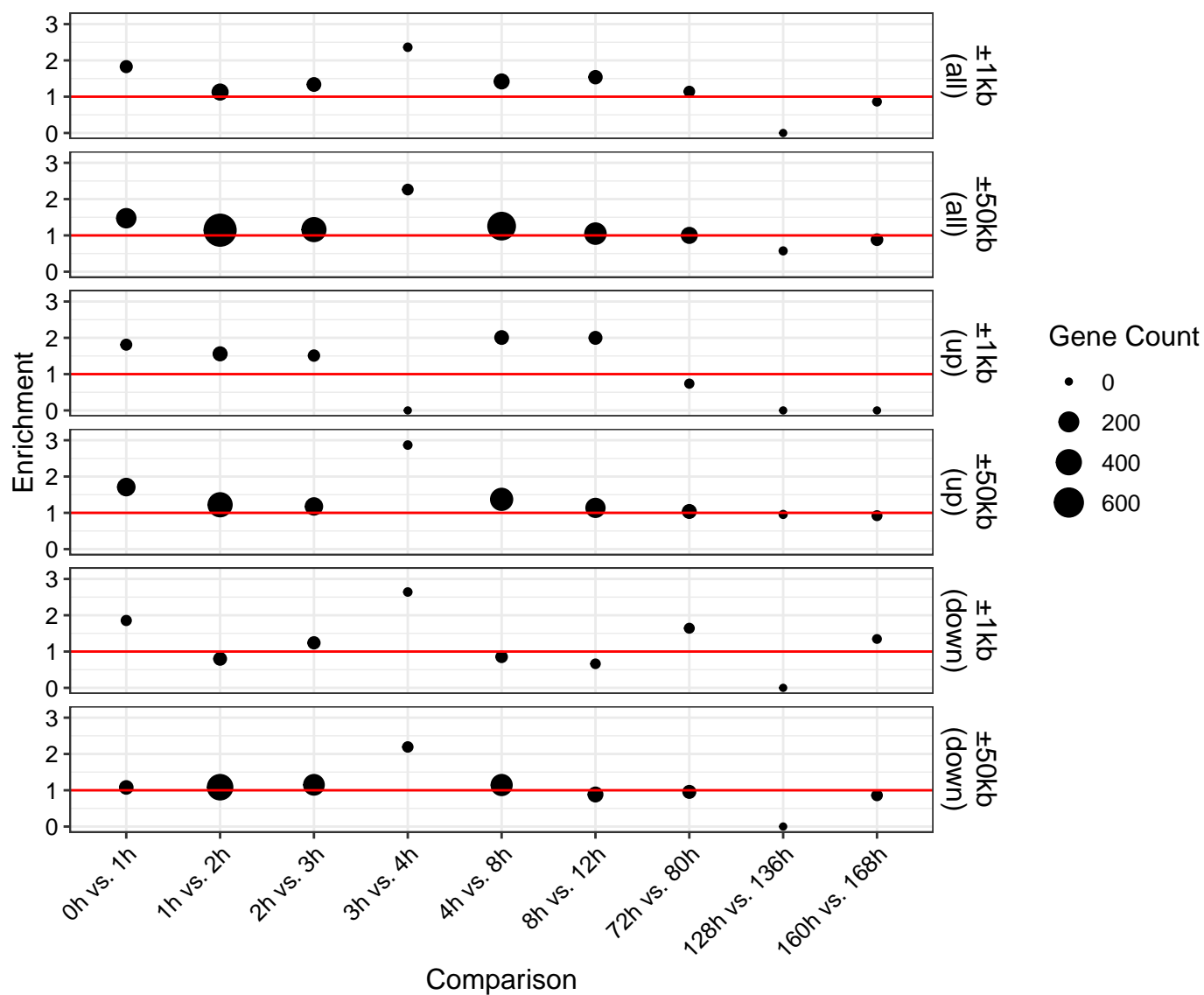

Supplement: S20 Fig — The enrichment of PU.1 binding to regions neighboring the DEGs between consecutive timepoints relative to the background set is plotted on the y-axis. The enrichment is plotted separately for all DEGs (“all”), upregulated DEGs (“up”), or downregulated DEGs (“down”) and for binding to proximal (±1kb) or distal (±50kb) regions. (PDF) [file pcbi.1014276.s020.pdf]

$\pm 1\text{kb}$

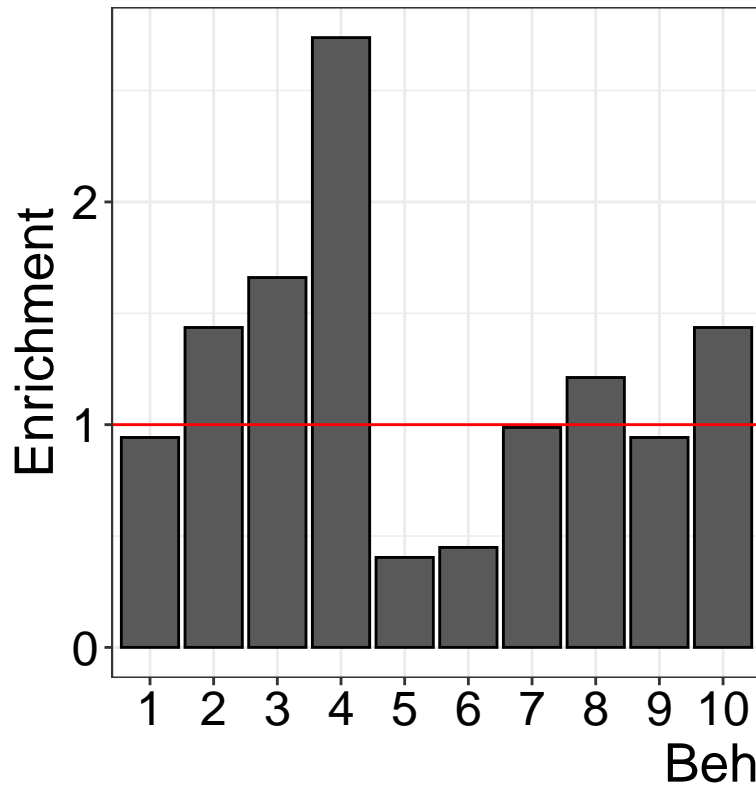

$\pm 50\text{kb}$

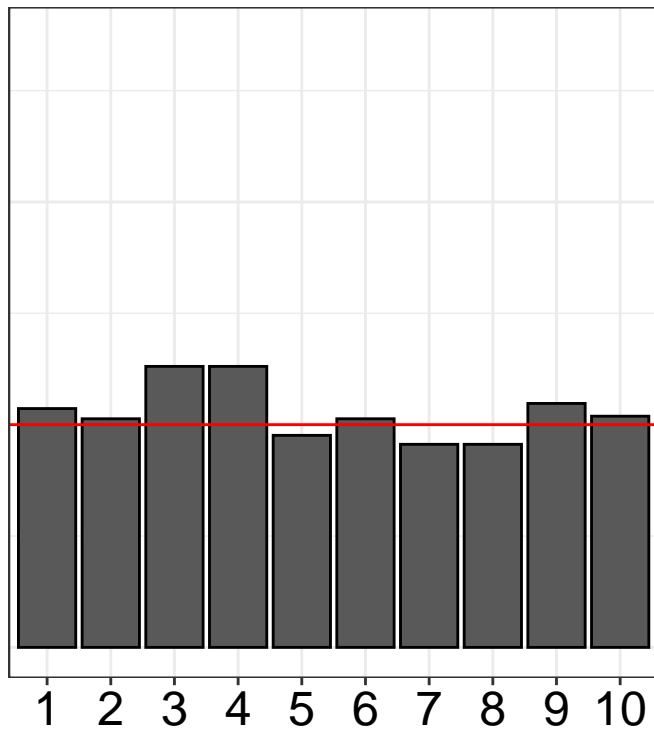

Supplement: S21 Fig — The enrichment of PU.1 binding to regions neighboring the top 500 genes for a behavior relative to the background set is plotted on the y-axis. The red line indicates no enrichment. (PDF) [file pcbi.1014276.s021.pdf]

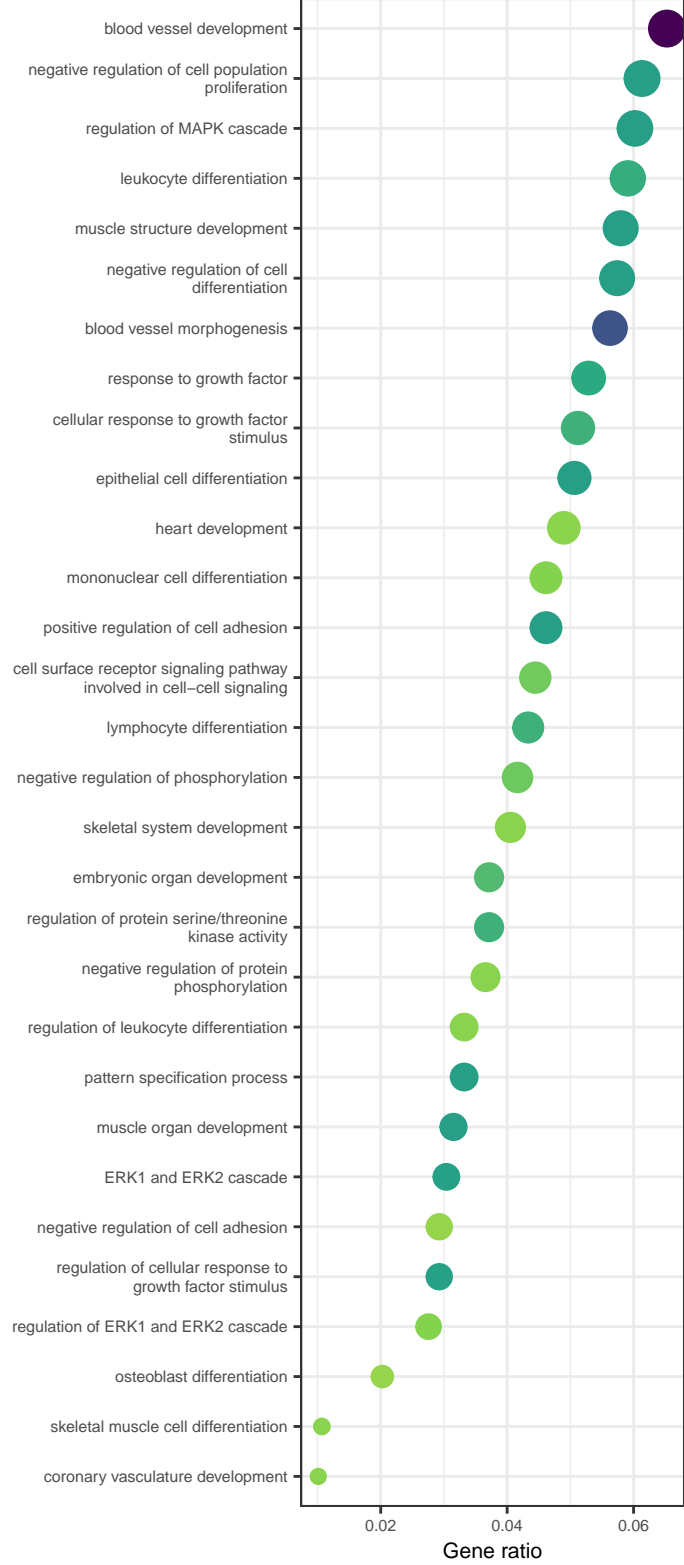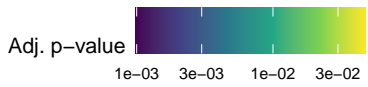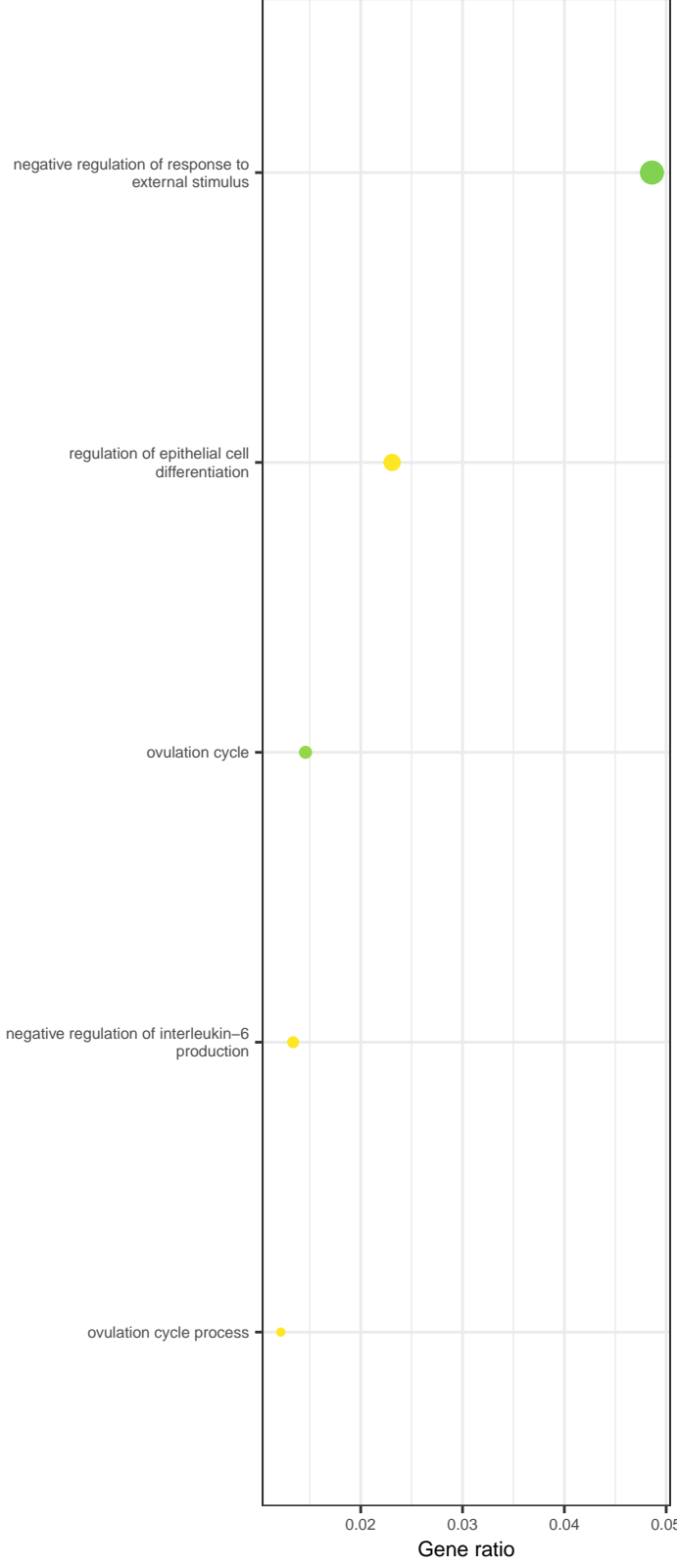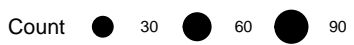

Supplement: S22 Fig — GO terms enriched for DEGs between 1h and 2h and between 2h and 3h are shown on the left and right respectively. See the legend of Fig 7 for plot description. (PDF) [file pcbi.1014276.s022.pdf]

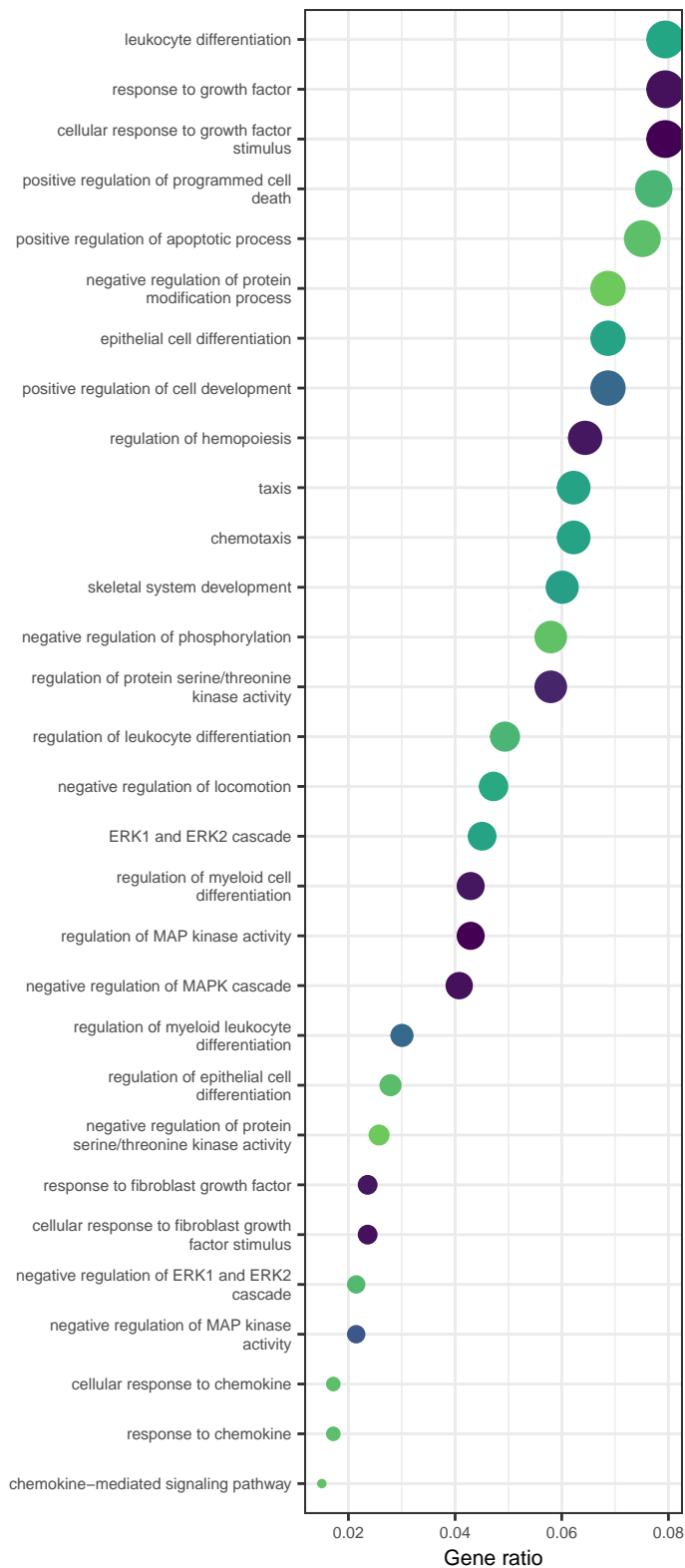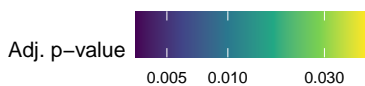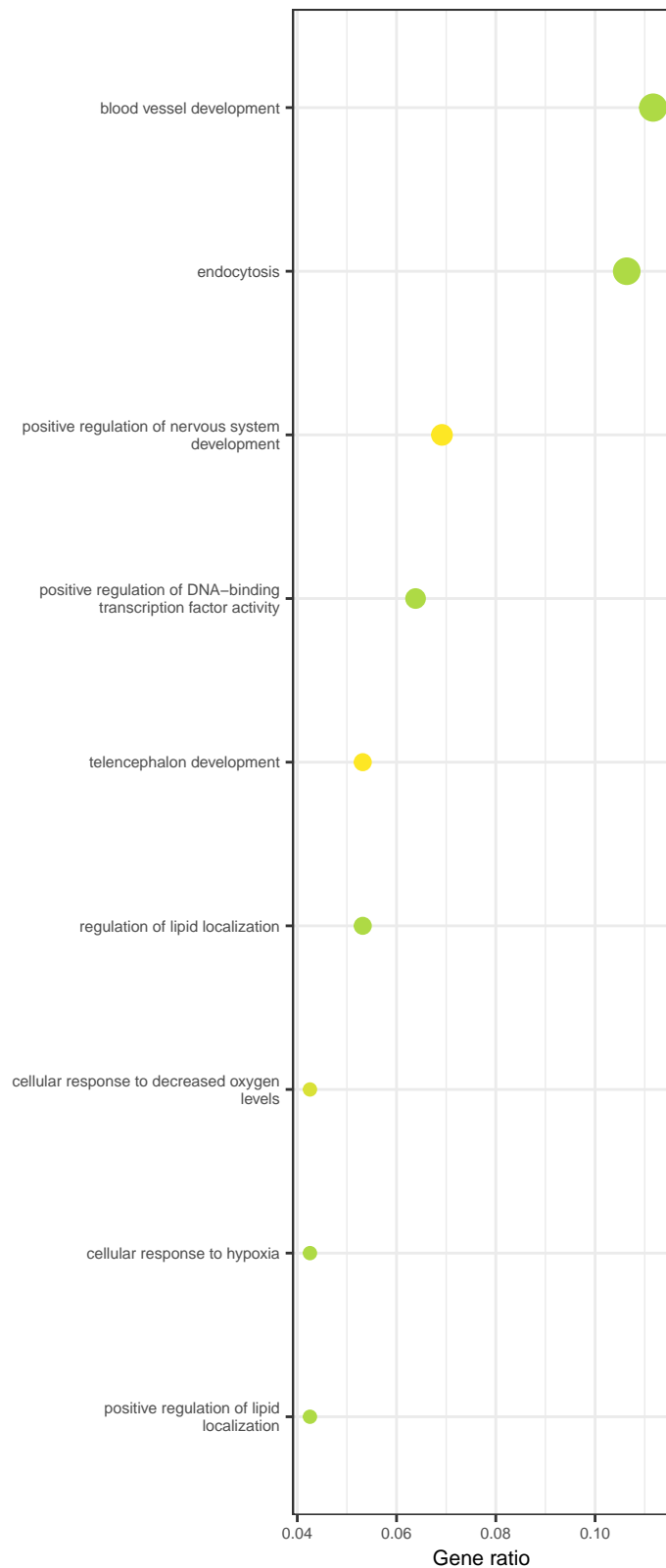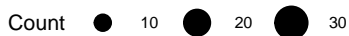

Supplement: S23 Fig — GO terms enriched for DEGs between 1h and 2h and between 2h and 3h are shown on the left and right respectively. See the legend of Fig 7 for plot description. (PDF) [file pcbi.1014276.s023.pdf]

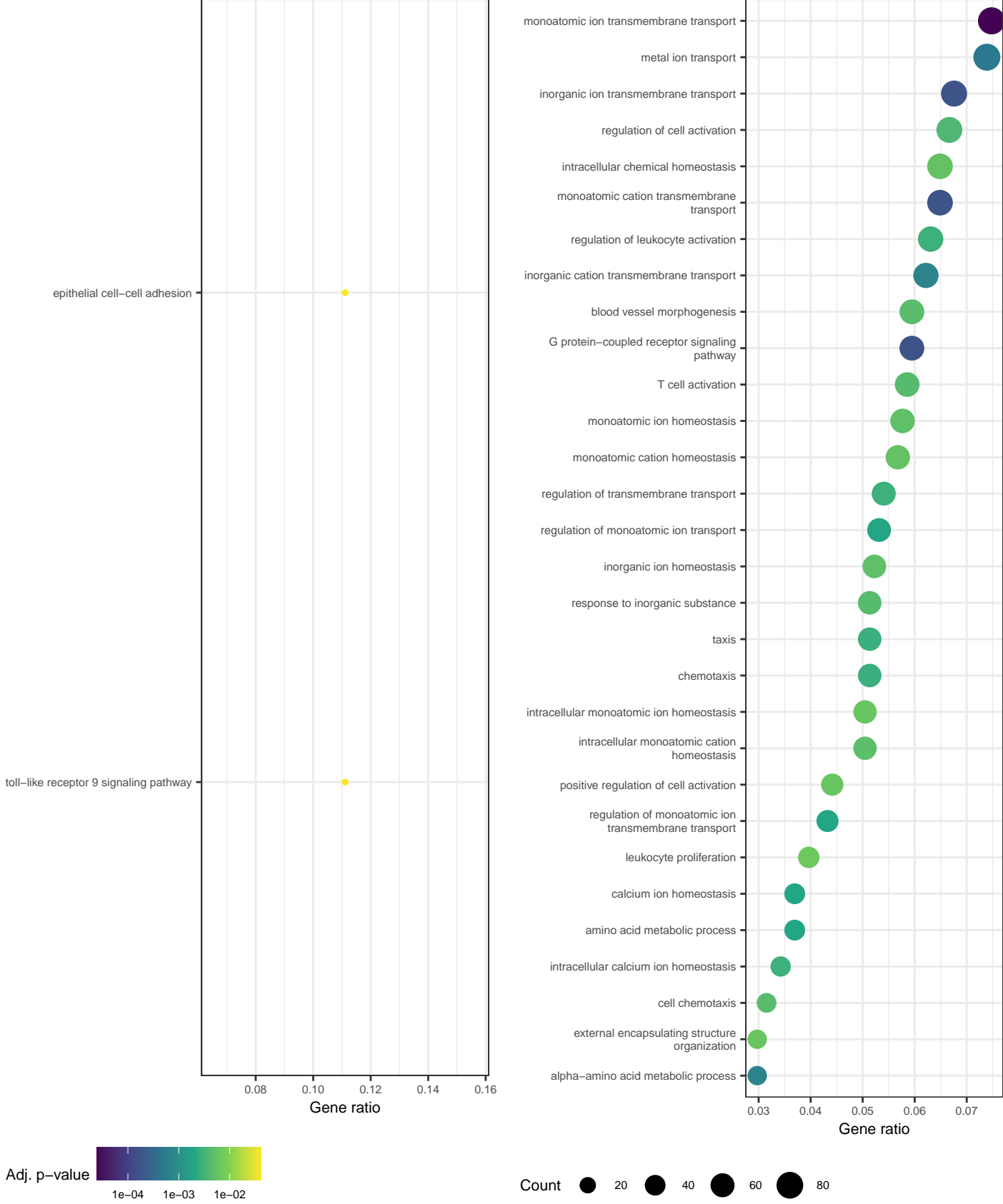

Supplement: S24 Fig — GO terms enriched for DEGs between 3h and 4h and between 4h and 8h are shown on the left and right respectively. See the legend of Fig 7 for plot description. (PDF) [file pcbi.1014276.s024.pdf]

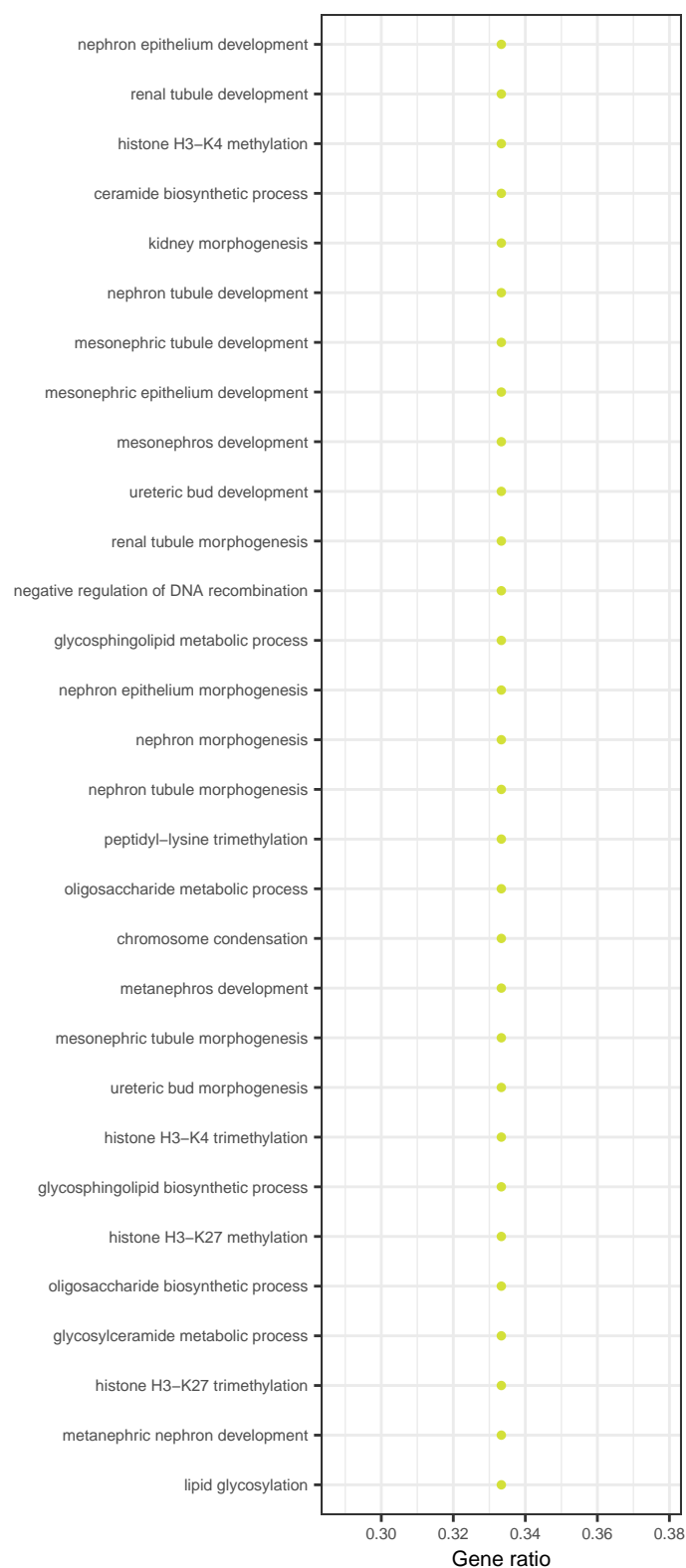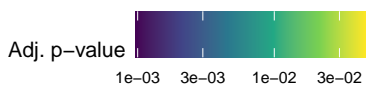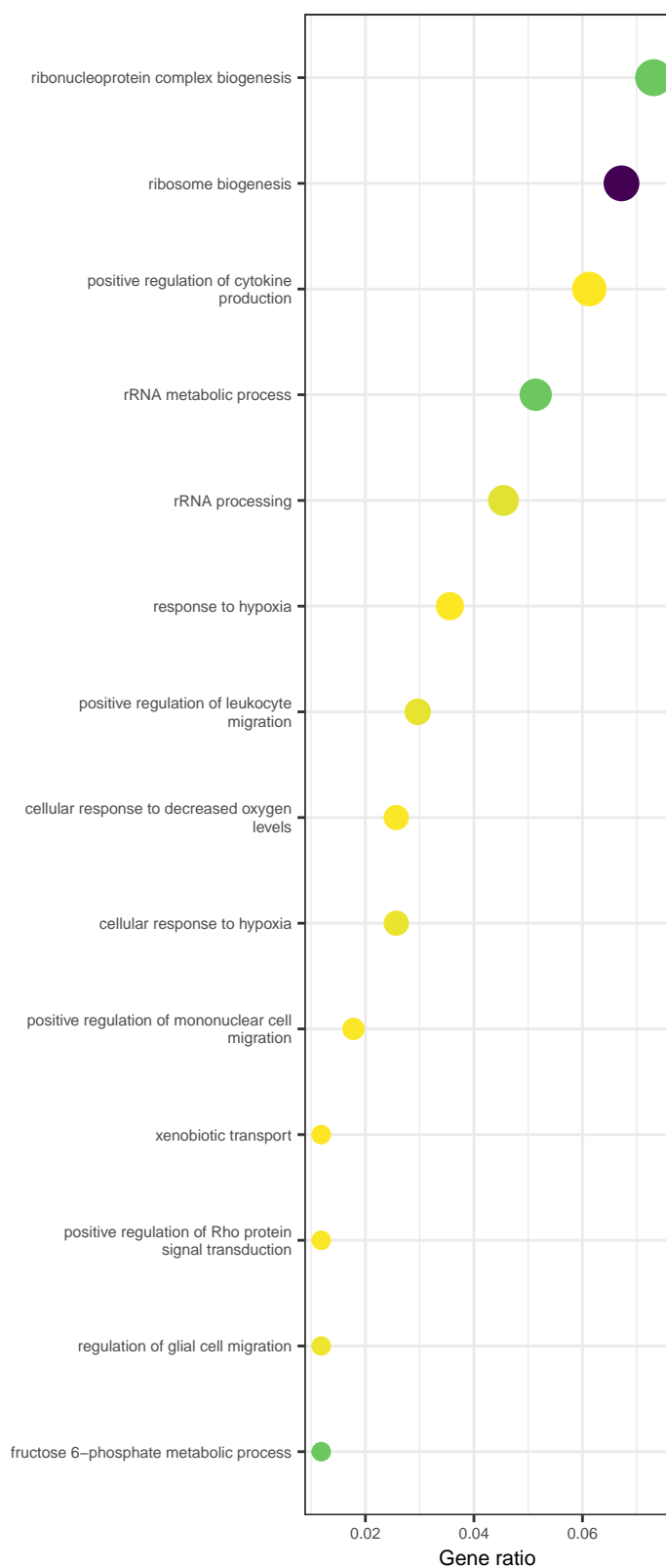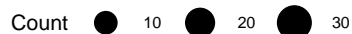

Supplement: S25 Fig — GO terms enriched for DEGs between 3h and 4h and between 4h and 8h are shown on the left and right respectively. See the legend of Fig 7 for plot description. (PDF) [file pcbi.1014276.s025.pdf]

# GO terms enriched for the top 500 Behavior 1 genes

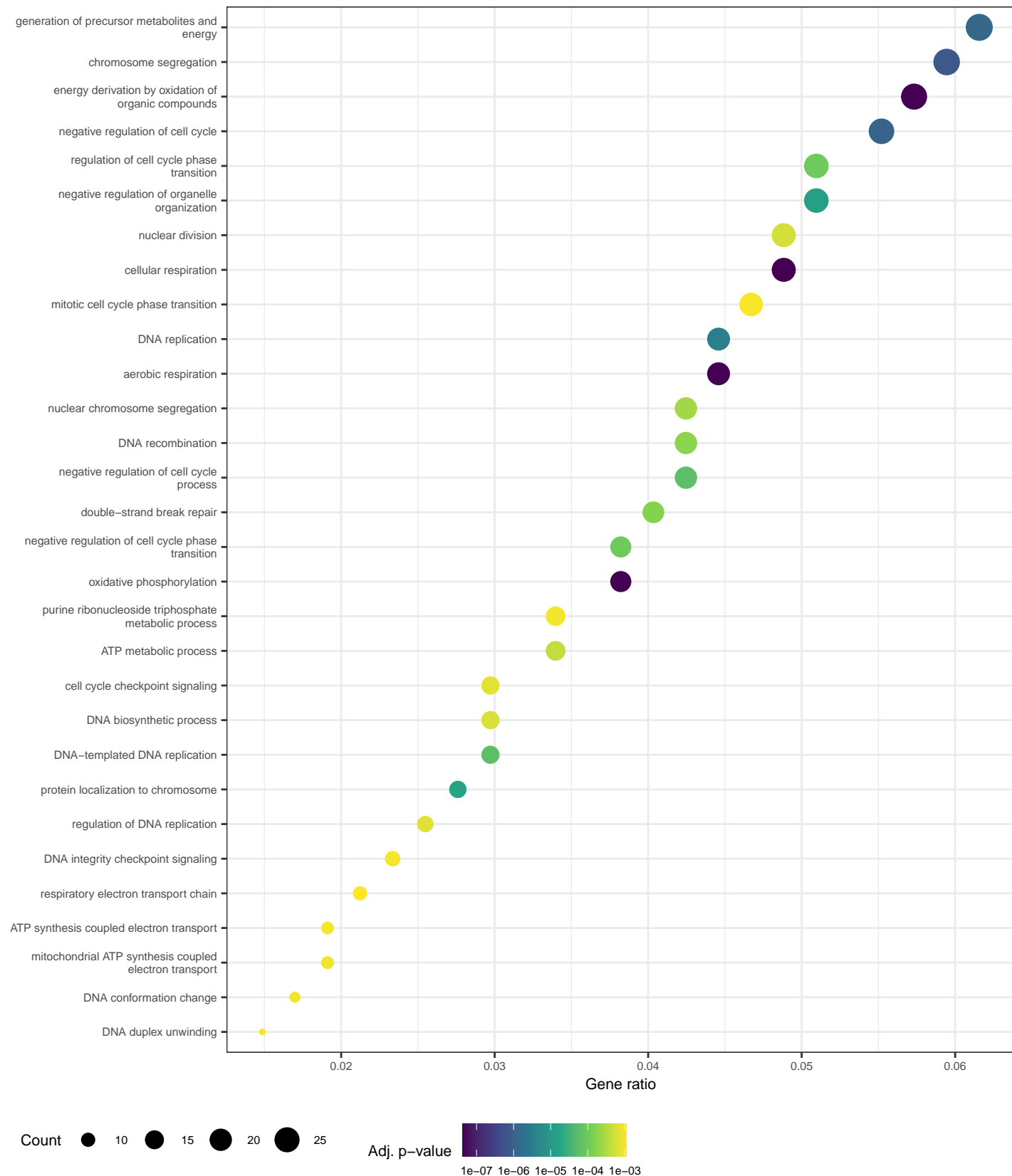

Supplement: S26 Fig — See the legend of Fig 7 for plot description. (PDF) [file pcbi.1014276.s026.pdf]

GO terms enriched for the top 500 Behavior 2 genes

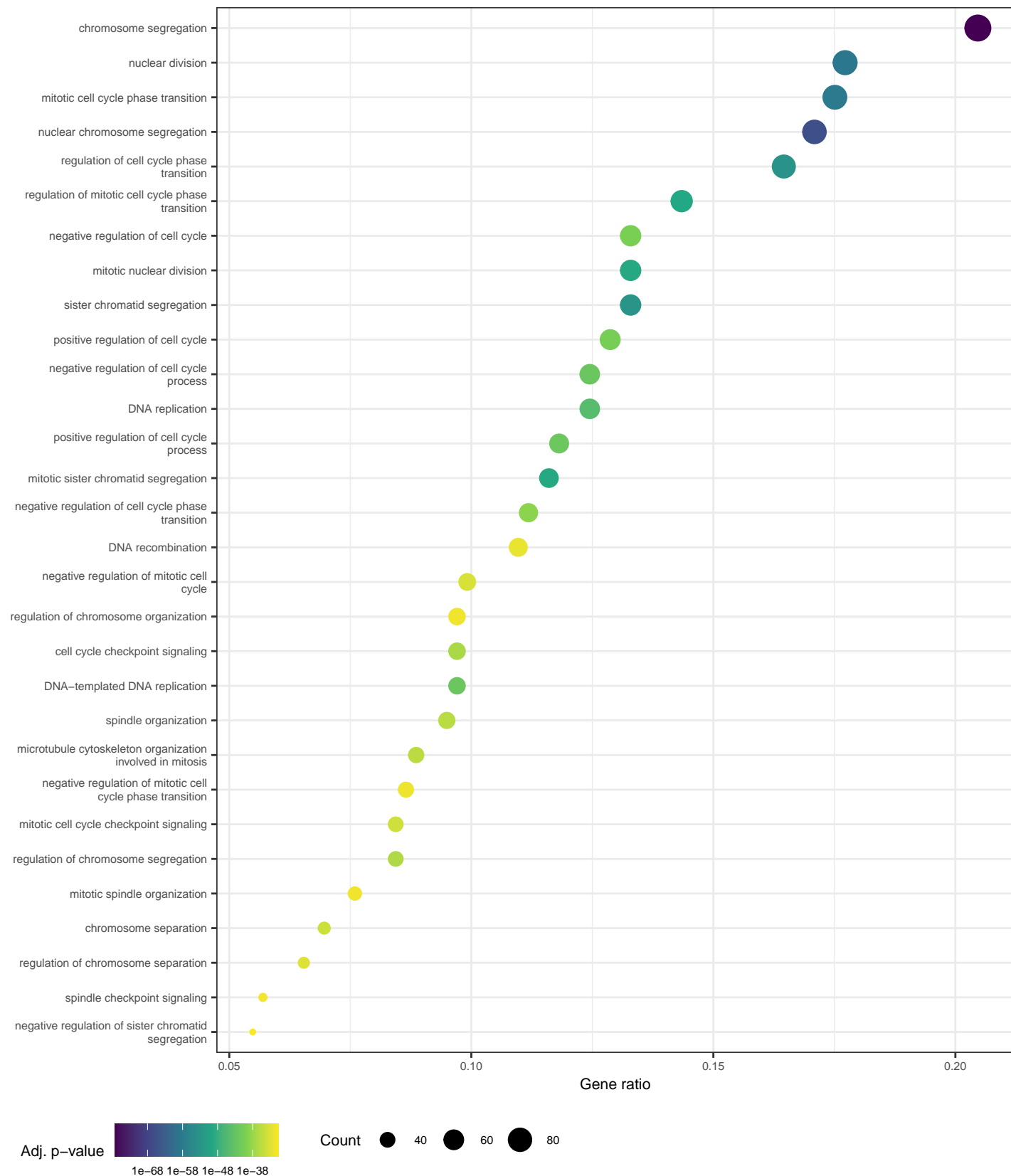

Supplement: S27 Fig — See the legend of Fig 7 for plot description. (PDF) [file pcbi.1014276.s027.pdf]

GO terms enriched for the top 500 Behavior 3 genes

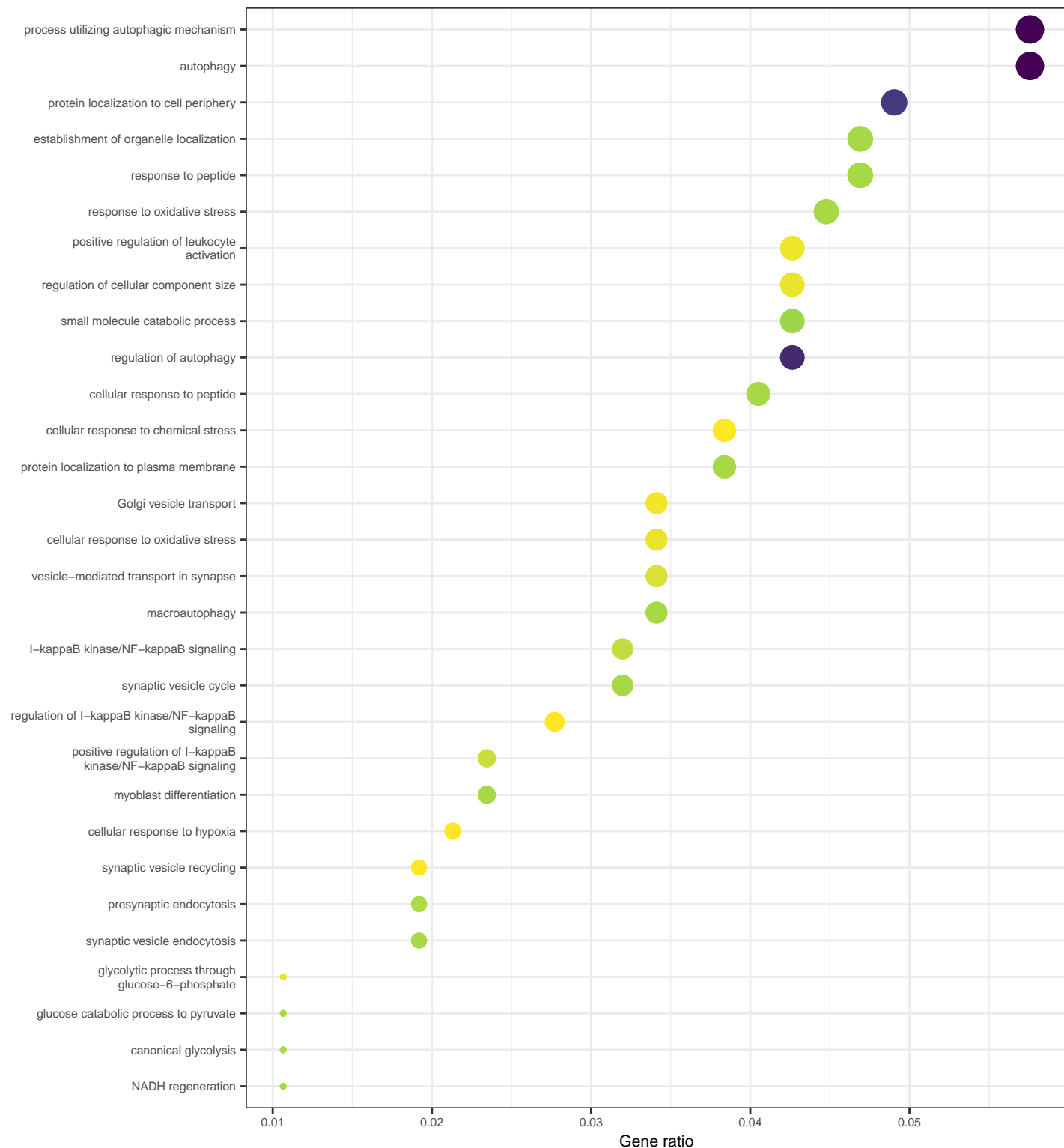

Count • 5 • 10 • 15 • 20 • 25

Adj. p-value

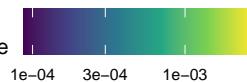

Supplement: S28 Fig — See the legend of Fig 7 for plot description. (PDF) [file pcbi.1014276.s028.pdf]

# GO terms enriched for the top 500 Behavior 4 genes

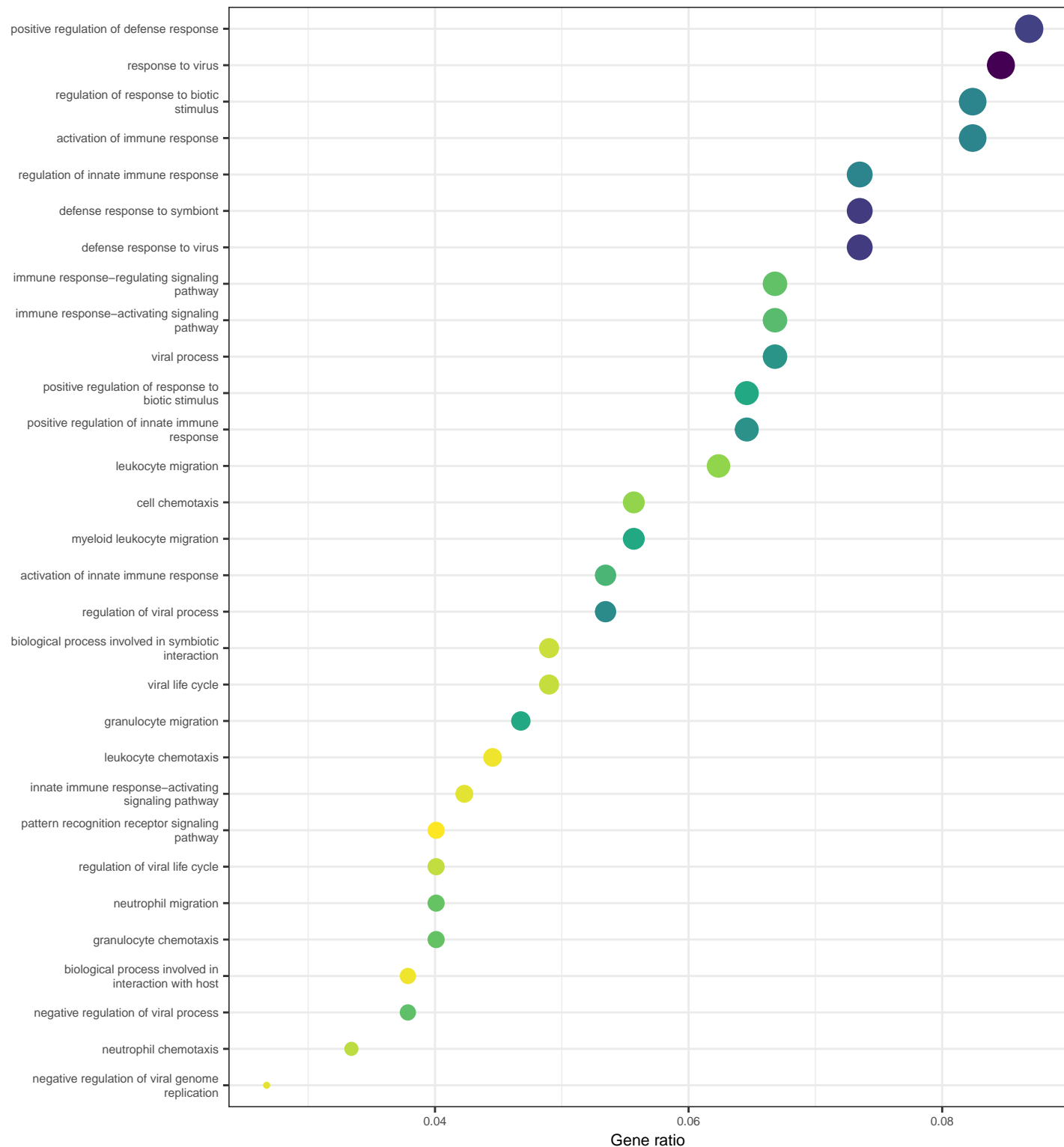

Adj. p-value

1e-13 1e-10 1e-07

Count 20 30

Supplement: S29 Fig — See the legend of Fig 7 for plot description. (PDF) [file pcbi.1014276.s029.pdf]

GO terms enriched for the top 500 Behavior 5 genes

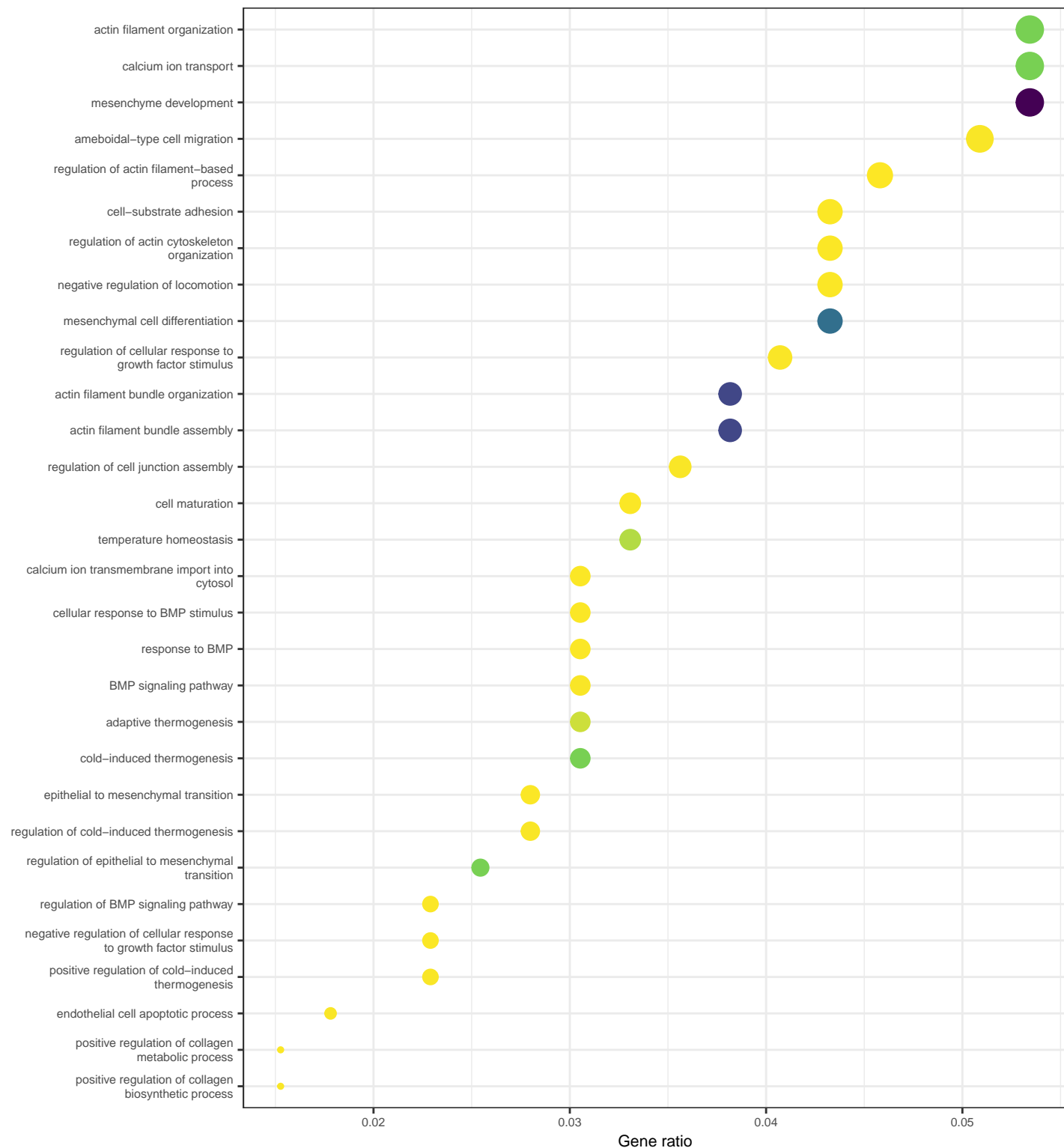

Count 10 15 20

Adj. p-value

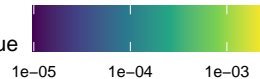

Supplement: S30 Fig — See the legend of Fig 7 for plot description. (PDF) [file pcbi.1014276.s030.pdf]

# GO terms enriched for the top 500 Behavior 6 genes

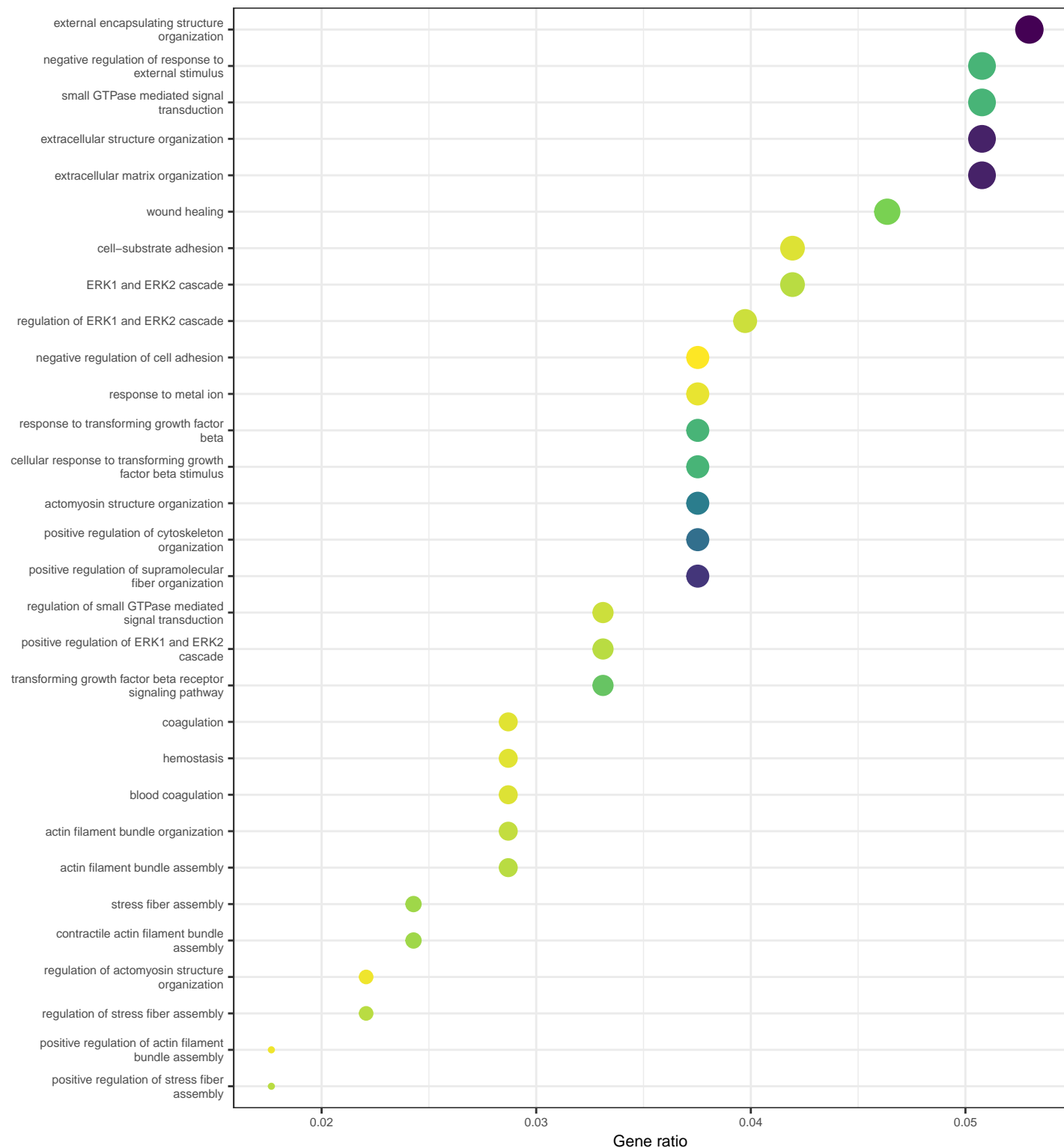

1e-05 1e-04 1e-03

Count 8 12 16 20 24

Supplement: S31 Fig — See the legend of Fig 7 for plot description. (PDF) [file pcbi.1014276.s031.pdf]

GO terms enriched for the top 500 Behavior 7 genes

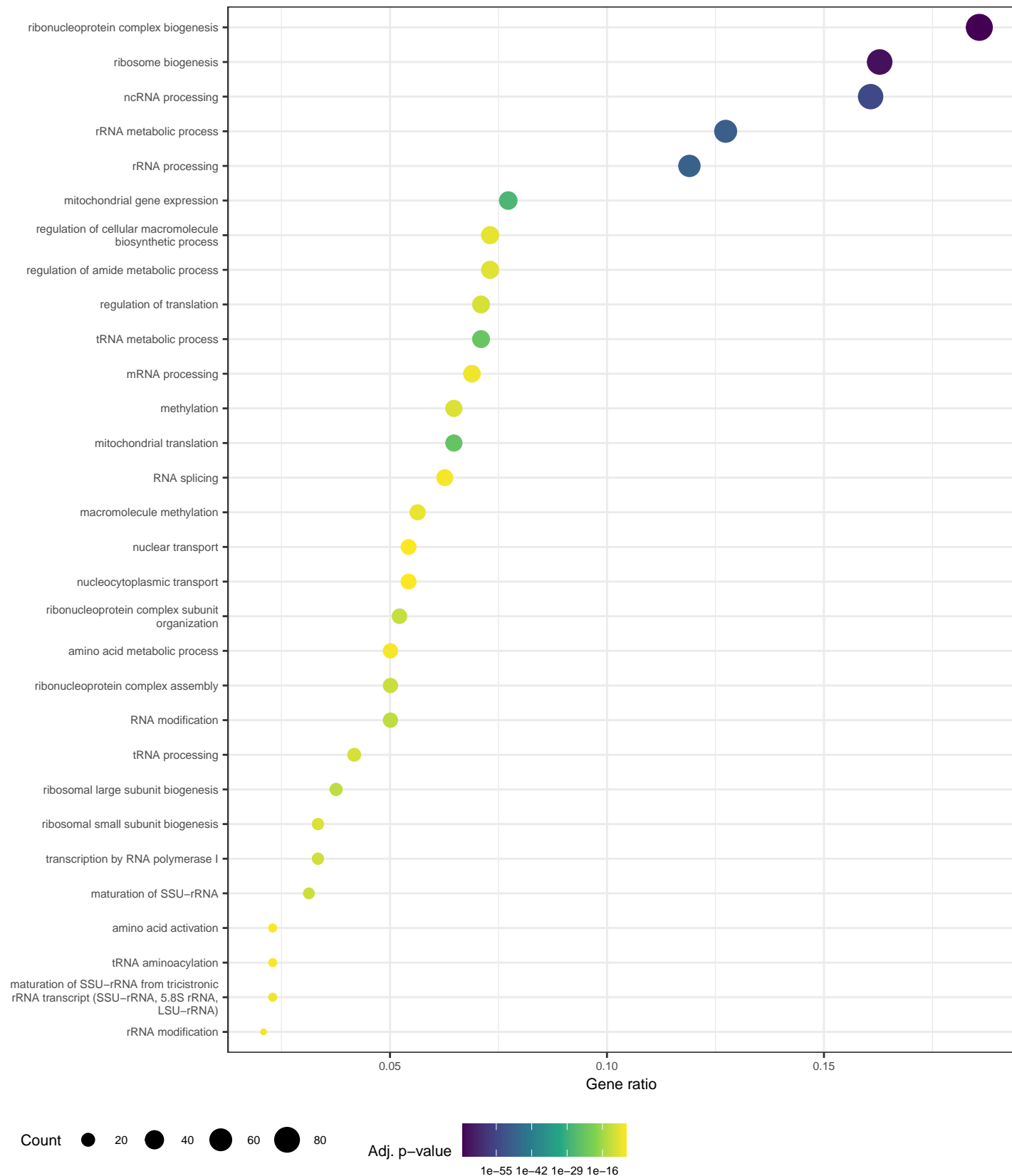

Supplement: S32 Fig — See the legend of Fig 7 for plot description. (PDF) [file pcbi.1014276.s032.pdf]

# GO terms enriched for the top 500 Behavior 8 genes

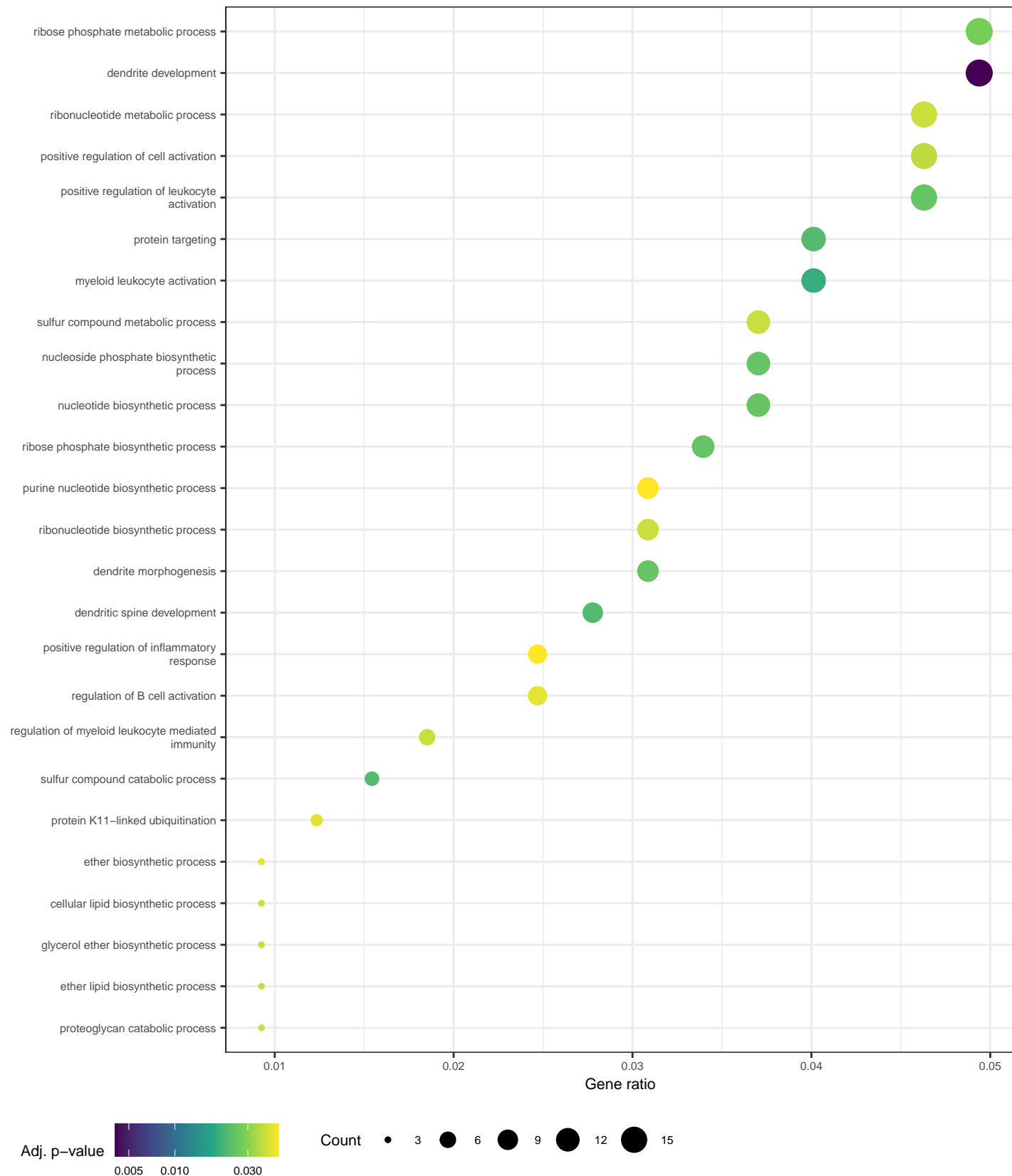

Supplement: S33 Fig — See the legend of Fig 7 for plot description. (PDF) [file pcbi.1014276.s033.pdf]

GO terms enriched for the top 500 Behavior 9 genes

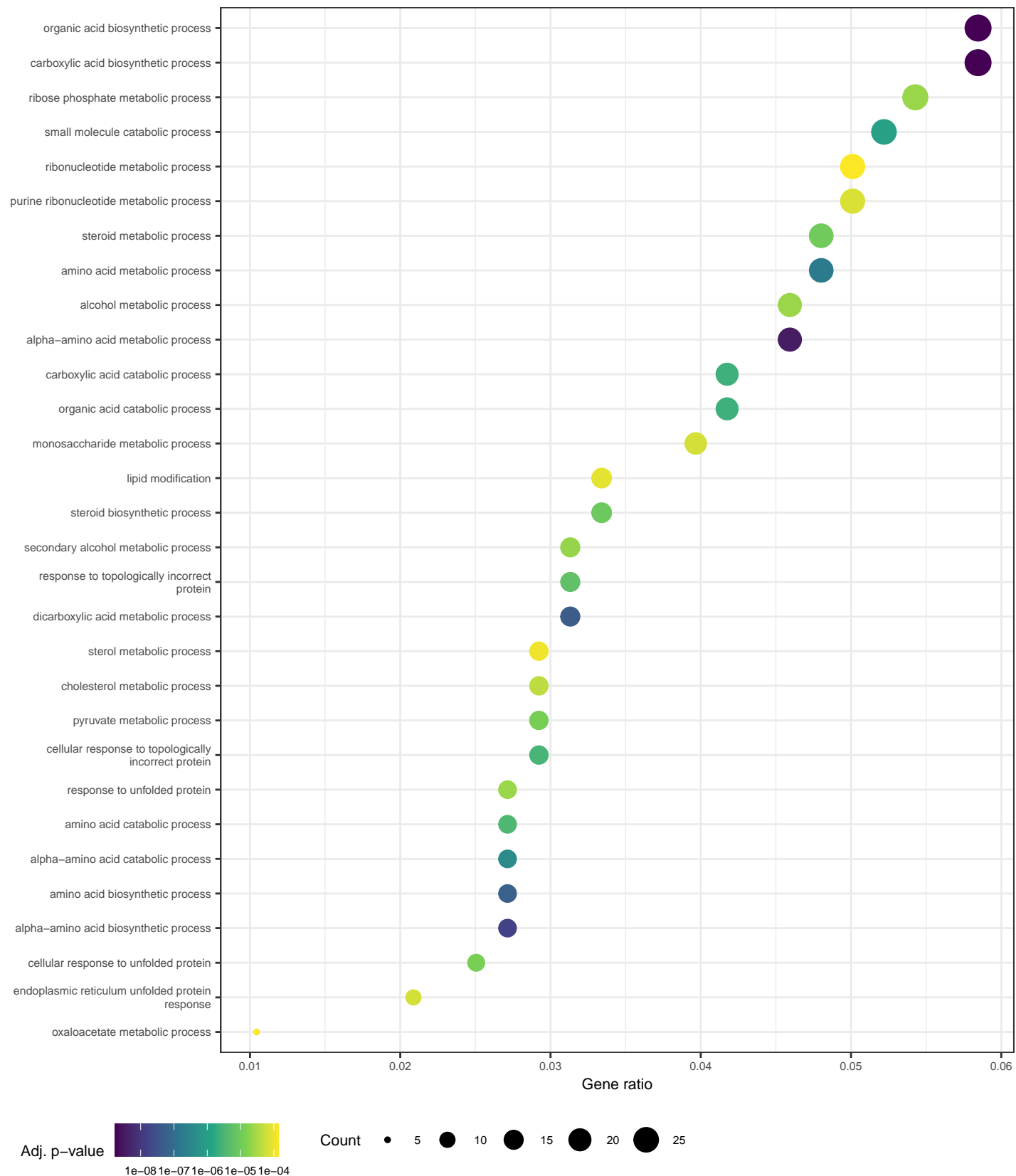

Supplement: S34 Fig — See the legend of Fig 7 for plot description. (PDF) [file pcbi.1014276.s034.pdf]

GO terms enriched for the top 500 Behavior 10 genes

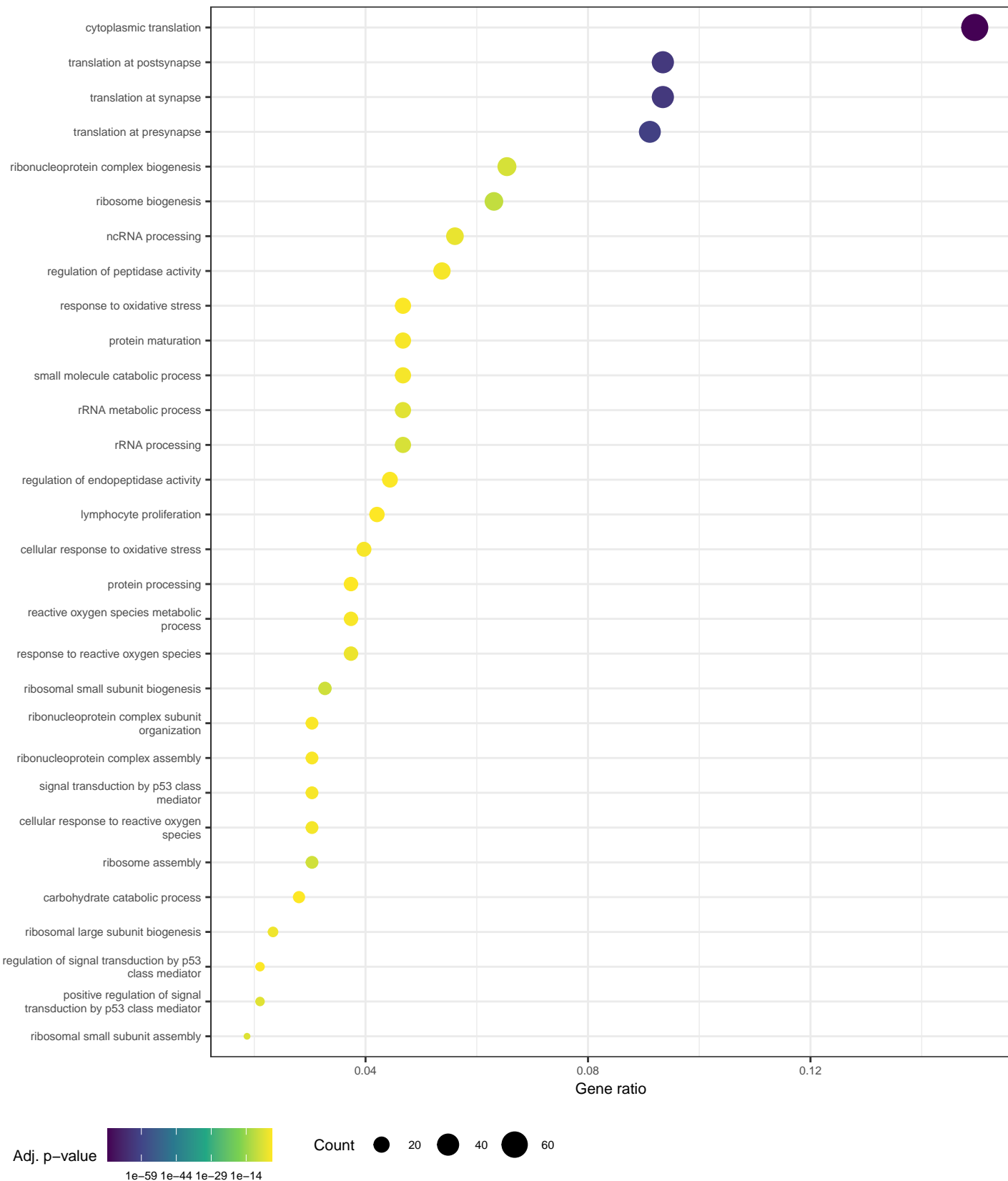

Supplement: S35 Fig — See the legend of Fig 7 for plot description. (PDF) [file pcbi.1014276.s035.pdf]

# Distance Matrix

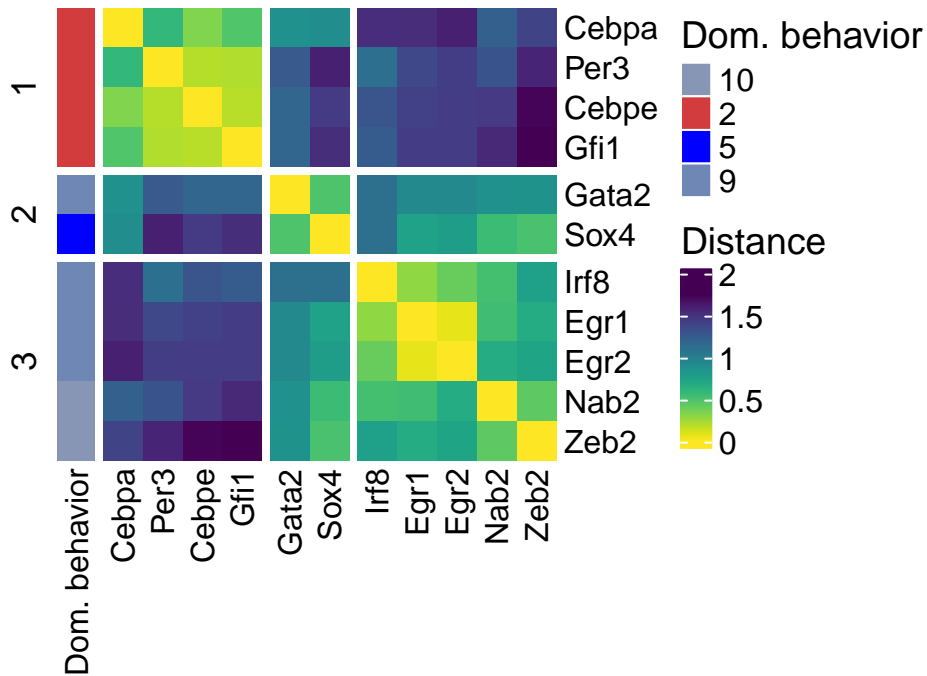

Supplement: S36 Fig — The similarity between the temporal expression of each pair of TFs, as measured by the distance 1 − r, where r is Pearson’s correlation coefficient is plotted as a heat map. Genes have been annotated by their behavior on the left. (PDF) [file pcbi.1014276.s036.pdf]

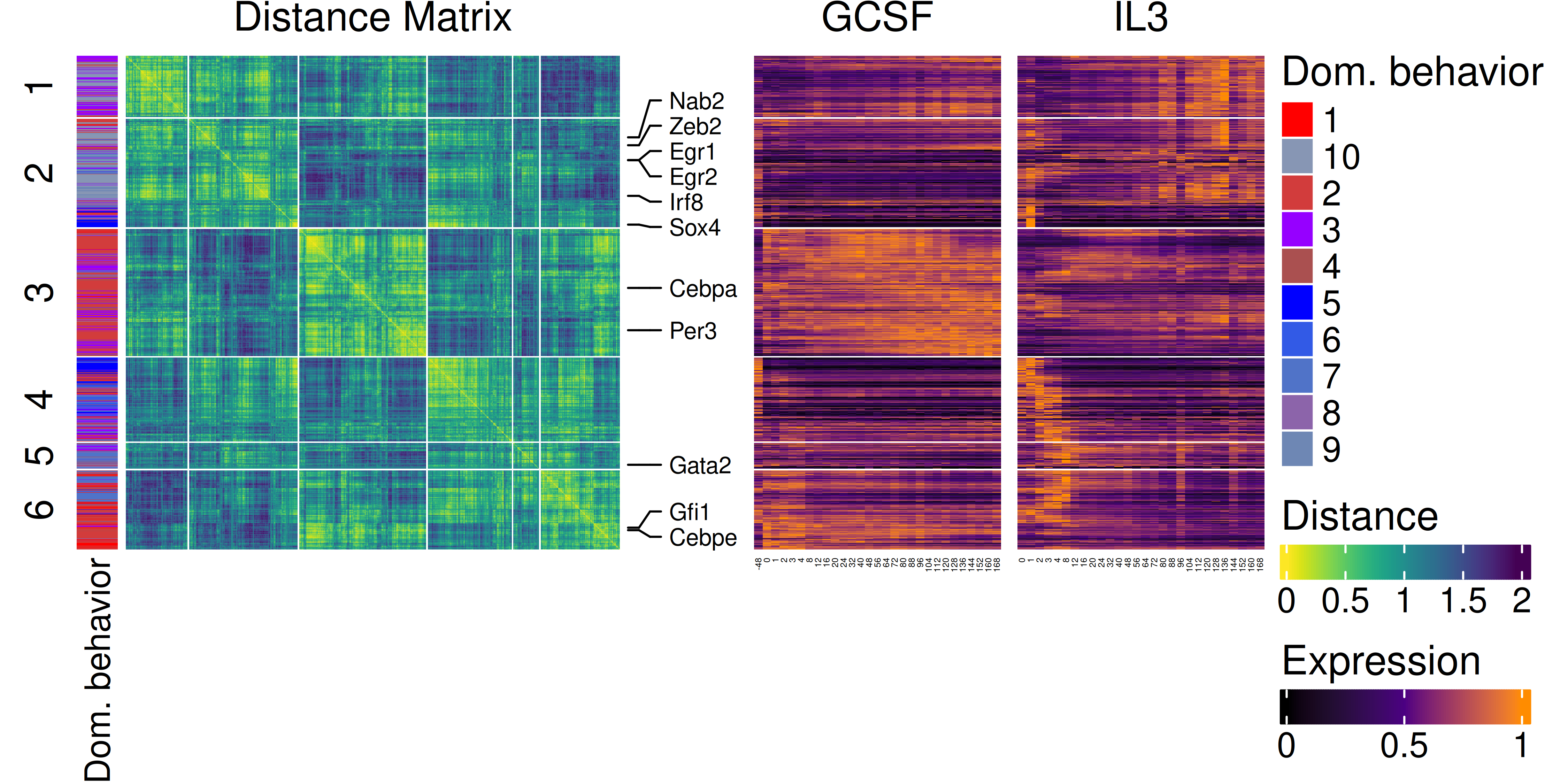

Supplement: S37 Fig — 2,331 TFs and cofactors were identified from TFCheckpoint2.0. TFs known to be involved in the macrophage-neutrophil decision are marked to the right of the distance matrix. See the legend of S16 Fig for the plot description. (TIFF) [file pcbi.1014276.s037.tiff]

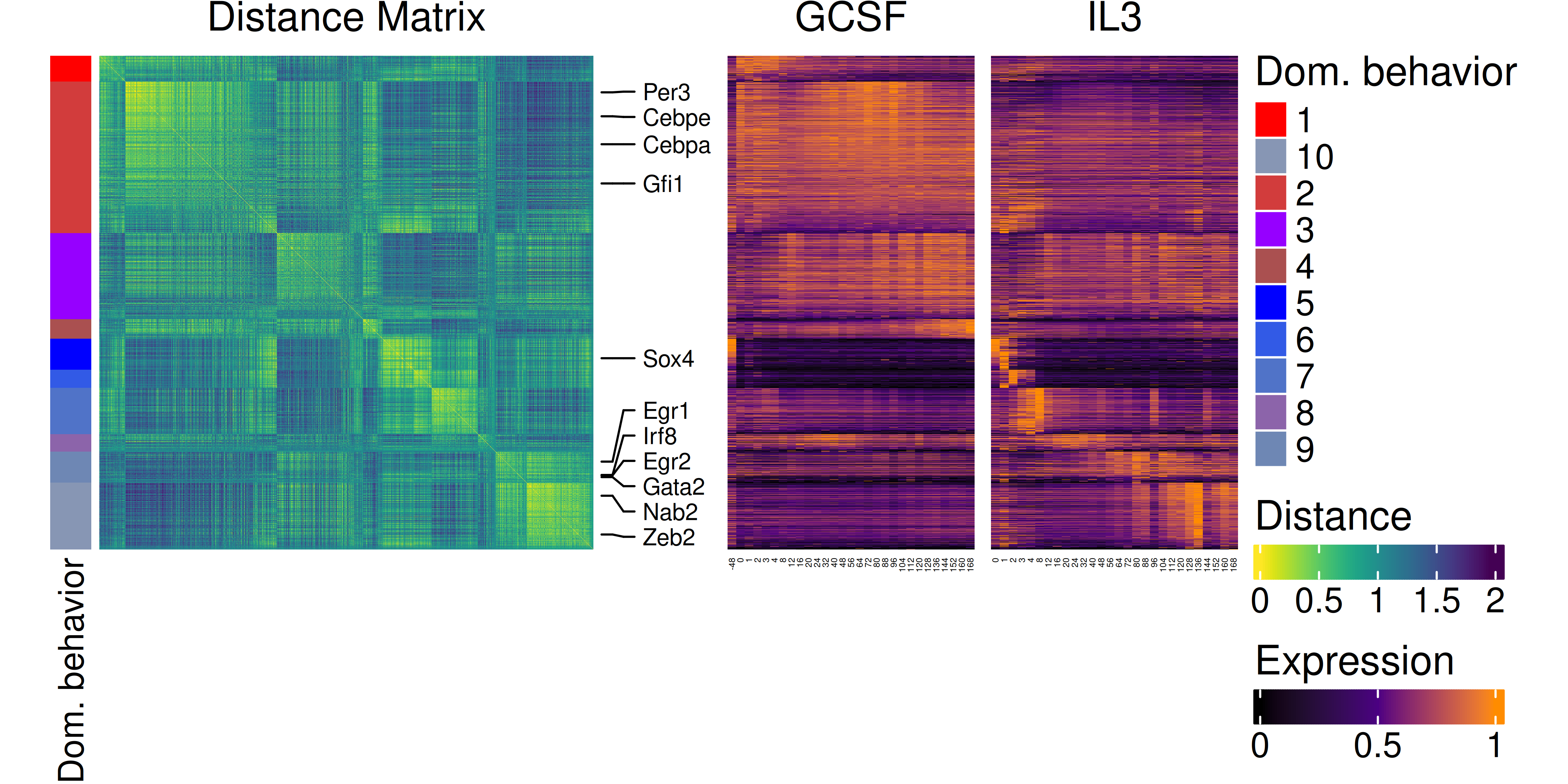

Supplement: S38 Fig — The TFs are ordered by their dominant behavior, annotated on the left. See the legends of S16 and S37 Figs for the plot description. (TIFF) [file pcbi.1014276.s038.tiff]
