# Supplementary material for: The differentiation of myeloid progenitors is effected by cascading waves of coordinated gene expression that remodel cellular physiology in a characteristic sequence
Source: PLoS Comput Biol. 2026 May 12;22(5):e1014276. doi: 10.1371/journal.pcbi.1014276 (PMC13189416; doi:10.1371/journal.pcbi.1014276)
Supplement: Attachment — Submitted filename: puerdiff_response_to_reviewers.pdf [file pcbi.1014276.s040.pdf]

Responses to reviewer comments on manuscript #PCOMPBIOL-D-21-01078

Overview: We thank the editor and reviewers for their insightful critiques. We have revised the manuscript extensively to address the critiques. We have performed the additional analyses suggested by all three reviewers and also the experiments suggested by reviewer 2.

In response to reviewer 1's comments, we have supplemented the comparison of PUER cells to *in vivo* bone-marrow (BM) cells in terms of global transcriptomic state with comparisons of known markers and TFs. The latter support the conclusions of the former well. We have also inferred GRNs from our time-series data, which recapitulate known GRNs, and showed that they are organized according to NMF behaviors.

Reviewer 2 questioned whether PUER differentiation truly models a bipotential lineage decision since GCSF pre-treatment might be irreversibly locking the cells into the neutrophil state. We have performed the "switch" experiment suggested by the reviewer and demonstrated that GCSF pre-treated cells remain bipotential. In response to the reviewer's suggestion to evaluate how PU.1 regulates genes in time, we generated a PU.1 CUT&RUN dataset and show that PU.1 binding is enriched in DEGs between consecutive early time points but not between consecutive late timepoints, showing that PU.1 tends to regulate early responses. The analysis of PU.1 binding enrichment in the top genes of each behavior showed that behaviors with early pulsatile and late sustained expression were enriched, implying that PU.1 also regulates terminal responses in addition to early ones.

Reviewer 3 questioned how static factors in NMF could model the highly dynamic patterns of gene expression observed in our time-series data and whether NMF would effectively cluster other data, specifically Tusi *et al.*'s dataset. We have explained below that all factors in NMF are not static, since the behaviors $H_m(t)$ vary in time. We have also applied NMF to Tusi *et al.*'s dataset and it was quite successful, inferring behaviors that correspond to the different lineages, and even intermediate developmental states, in an unsupervised manner without any prior knowledge of marker or TF identity.

Both reviewer 1 and 3 raised concerns about our decision to scale the maximum expression of each gene to 1 before performing NMF. We have addressed this in two ways. First, we have shown that NMF on unscaled data results in substantially similar behaviors as NMF on scaled data. Second, we have shown that NMF on Tusi *et al.*'s unscaled data performs very poorly and does not infer most of the lineages.

A point by point description of the changes follows. *Note:* New supplementary figures added and the line numbers of revised text are highlighted in red below.

Major points

Reviewer 1

1. The comparison of the in vitro differentiated cells to in vivo data utlizes global transcriptome-wide metrics to measure the distance between different cell populations. However, this

analysis should be complemented by analysis for the regulation of specific genes / gene programs that may be associated with progenitor or differentiated states. For instance, what macrophage-specific genes to OHT treated PUER cells express or fail to express? Similarly, are there stem/progenitor associated programs that are expressed or not prior to induction of differentiation?

We have supplemented our analysis of global transcriptomic state between PUER cells and bone-marrow data with comparisons of individual lineage marker and TF genes (Figs. S6–S11). These comparisons are in agreement with the global analysis and the general understanding of myeloid differentiation. The markers and TFs by and large behave as one would expect. Described on lines 165–195.

2. Fig. 4: How are the cutoffs for the genes to b showed for the NMFF determined? The authors select this be the top 200 genes, but at what point down the list do these weights become negligible? Relatedly, are the red lines indicative of the weighted average of the behaviors? They do not seem anywhere close to being an average of the top of 200 genes with differential behaviors.

The red lines are not weighted averages, but the temporal expression pattern ("behavior") of each metagene ($H_m(t)$). The individual gene expression patterns are shown to illustrate the correspondence between the behaviors and the expression patterns of individual genes. We chose the genes with the highest 200 weights so that we show sufficient examples without making the panels too busy. The goal here is to show that individual genes exhibit the temporal expression pattern of the behavior. However, an exact correspondence between individual genes' patterns and the behavior's patterns is not expected. Many genes have high weights for multiple behaviors so that their expression pattern is a weighted sum of multiple behaviors.

This has two consequences. First, we expect behaviors to match parts of individual genes' expression patterns but not the whole pattern. Second, we expect genes to display less extreme changes than the behaviors themselves. This accounts for the reviewer's observations below about behaviors 1 and 3 in the IL3-OHT condition. Behavior 3 genes in IL3-OHT conditions undulate in the same pattern as the behavior, showing its contribution. However, individual genes' expression doed not go to zero when the pattern does so due to contributions from other behaviors. Similarly, the non-zero but constant (in time) expression of top behavior 1 genes is due to multiplication of behavior 1 expression by its weight and contributions from other behaviors.

In order to better illustrate the relation between behaviors and individual gene expression patterns, we have added additional panels to Figure 4 that reconstruct the temporal expression pattern of *Cx3cr1* as an example by sequentially accumulating weighted contributions from each behavior.

3. Related to the point above: the NMFF groupings appear to highlight temporal differences while underplaying initial baselines or fold differences. This is concerning because though many behaviors appear to show a clear, transient pulse on average, some of the gene expression changes appear to look much more gradual. (e..g this is especially prominent for Behaviors 1 and 3 for 4-OHT only treated cells, where there is sustained expression of many genes that appear to be outside the time window of the pulse as indicated by the red lines). Because of these concerns, it would be important to (1) use a complementary method to group together genes with similar expression time course, and (2) perform

this analysis not only on z-score normalized traces, but on raw traces or traces where no normalization for baseline / effect size is performed.

(1) We have clustered genes hierarchically to check correspondence with the NMF analysis (Fig. S17). The groups of temporal expression patterns identified by hierarchical clustering are very similar to the behaviors. Annotating each gene by its dominant behavior—the one with the highest weight—shows that a majority of the genes in a correlated group have the same dominant behavior. Therefore, there is good agreement between NMF and hierarchical clustering. Furthermore, grouping genes merely by dominant behavior reveals strong block diagonal structure (Fig. S16), showing that the dominant behavior alone can account for the observed correlations to a large extent. Line numbers 422–433.

(2) We have also performed NMF on the original unscaled data (Fig. S15). All the behaviors inferred from scaled data are present showing that scaling does not adversely affect behavior inference. Despite this result, we believe that scaling gene expression before performing NMF is the superior approach. Given the long-tailed distribution of RNA-Seq data, NMF (and other clustering algorithms) would be dominated by a relatively small number of highly expressed genes if the data were not scaled and the full diversity of expression patterns would be missed by the analysis. This is most apparent in our application of NMF to Tusi *et al.*'s scRNA-Seq data (Fig. S18–S19) in response to a similar comment by reviewer 3 (please see below). Line numbers 369–371.

We would also like to clarify that we did not perform z-score normalization but instead scaled the maximum expression of each gene to 1; non-zero minimum expression remained non-zero after the scaling. We have made this clearer in the revision.

4. Generally, there is very little further analysis of transcriptomic changes in relation to specific genes or gene regualtory networks important for Gr or Mac differentiation. Fig. 7 and Fig. 8 show gene ontologies but these do not highlight or establish specific regulators or candidates.

We have significantly expanded our analysis to 1) analyzing individual marker and TF genes (Fig. S6–S11), 2) using co-regulated TFs to infer GRNs (Fig. S36–S38 and line numbers 611–647), and 3) analyzing PU.1 binding in relation to DEGs and behaviors in response to a comment by reviewer 2 (Fig. S20–S21 and line numbers 487–524). The key takeaways from the inferred GRNs is that the TFs known to coordinate macrophage-neutrophil differentiation observe the same regulatory relationships in PUER cells as observed in prior work (Fig. S36) and that the core GRNs are embedded in much larger GRNs formed by genes exhibiting similar behaviors.

Reviewer 2

1. 1. In the introduction, the authors discuss how progenitors with more than one possible cell fate might be biased toward one fate or another by expression of a key regulator/TF. PU.1 is such a key regulator, and PUER cells provide a model for using a single TF to trigger differentiation. However, the samples designated as 0 hours have been cultured in different conditions (either IL-3 or 48h of GCSF), and the authors make it clear that there are many gene expression changes during the 48h pre- treatment with GCSF, and that the two 0h datapoints differ markedly in their gene expression (e.g. the PCA in Fig 2C, Fig 3C and also shown by Behavior 1 in fig 4; Fig S6A). In line 56 of the introduction, the authors state that

GCSF can initiate fate transformation in PUER cells. It is therefore unclear to me whether the authors think that the early gene expression changes they measure are an immediate cause/consequence of lineage choice of bipotent PUER cells, or if they are capturing the early stages of differentiation triggered by PUER activation in the contexts of macrophage- and neutrophil-biased (or indeed committed) cells. In the author summary, they refer only to cell "maturation", but elsewhere refer several times to lineage decisions: Line 384 - ..."may indicate important moments when crucial lineage decisions are made"; line 413 "...that the cell fate decision is made very early during the differentiation process"; line 431 "...that the cell-fate decision is made within the first 4 hours of OHT induction". And in the discussion - line 569 "...that the cell-fate decision appears to have been made by the time of the 8 – 12h transition". I agree with this final statement, but it is not clear to me that the timeseries has captured gene expression data from cells before or during the point that the cell fate decision was made. This could easily be explored by testing the lineage potential of the PUER cells pre-treated with GCSF for 48h, by switching them back into IL-3 when (or before) 4-OHT is added. Cell potential could be assessed by morphology of the differentiated cells, and/or by gene expression analysis of some key genes that distinguish between neutrophil and macrophage lineages. If the authors can demonstrate that most of the GCSF pre-treated cells have retained macrophage potential, this would indicate they have indeed analysed the neutrophil/macrophage lineage decision point, which would strengthen the paper. If the authors do not wish to address this experimentally, they should clarify the evidence that the PUER cells remain bipotent in both conditions at the timepoint they designate 0h, or re-assess their use of terms referencing lineage decisions.

The reviewer questions whether PUER differentiation truly models a bipotential lineage decision since it is possible that GCSF pre-treatment irreversibly commits cells to the neutrophil fate. In that scenario, our earliest timepoints after OHT induction would not be profiling the lineage decision, which would have been made during the GCSF pre-treatment period. We have carried out the experiment suggested by the reviewer and the results clearly show that GCSF pre-treated PUER cells retain macrophage/monocyte potential (Fig. S12 and S13 and line numbers 209–220) and therefore our earliest timepoints are indeed documenting the implementation of the lineage decision. We carried out PUER differentiation in IL3+OHT and GCSF+OHT as usual but included an additional "switch" condition. In the switch sample, cells were pre-treated with GCSF for 48 hours but were switched back to IL3 prior to OHT induction. The differentiation was characterized by flow cytometry and Wright-Giemsa stain and the switch sample is almost identical to the IL3-OHT sample, showing that GCSF pre-treated cells remain bipotential until OHT induction.

2. 2. Related to the point above, if the authors do undertake biological experiments to address cell potential, it would be interesting to know to what extent the differentiation and associated gene expression changes are dependent on continuous activation of PUER (by the addition of fresh OHT at 40h, 88h and 136h), or whether OHT can be withdrawn after the 8h or 80h transition points.

While these additional experiments would be important to further characterize at what point the decision is rendered irreversible, our results, conclusions, and interpretations do not depend on their outcome. We hope that the reviewer will agree that these experiments belong in future studies.

3. 3. Although the manuscript primarily concerns genome-wide changes in the transcriptome and how these relate to cell state, the changes are being triggered by experimental manip-

ulation of a single transcription factor. The authors discuss the possibility of conducting "multi-omic" analyses in the future to clarify the causality of events (line 574). It is surprising to me that the authors have not utilised pre-existing ChipSeq datasets for PU.1 and/or PUER in hematopoietic cells to provide some insight into which – or what proportion of - the differentially expressed genes are likely to be directly bound and regulated by PUER, and whether these are concentrated in particular gene expression behaviors. For example, genes displaying Behavior 8 (upregulated between 0h and 80h in both GCSF and IL3) seem likely to be highly enriched for direct targets of PUER. Although there are obvious reservations to using binding or chromatin accessibility data taken from different cell types/models, this should be explored.

We have conducted the analyses suggested by the reviewer. We generated a PU.1 CUT&RUN dataset in undifferentiated and 48h IL3-OHT PUER cells to ensure that TF binding and gene expression datasets were compatible. We then checked the enrichment of PU.1 peaks near the TSS or in distal regions of 1) DEGs between consecutive timepoints or 2) the top 500 genes in each behavior. We found that while DEGs between early consecutive timepoints are significantly enriched for PU.1 binding near the TSS and also in distal regions, DEGs between later consecutive timepoints are not (Fig. S20 and line numbers 487–527). This shows, perhaps not unexpectedly, that genes modulated early are likely to be direct PU.1 targets. Also, downregulated genes show little to no enrichment of PU.1 binding, showing that PU.1 primarily acts as an activator. Behavior 3, that is upregulated early in both GCSF and IL3 conditions as well as the late behaviors for GCSF and IL3, 4 and 10 respectively, are enriched in PU.1 binding, showing that PU.1 regulates both initial and sustained responses. In contrast, the GCSF pre-treatment responsive behavior, behavior 1, the down-regulated behavior, behavior 5, are not enriched in PU.1 binding (Fig. S21).

Reviewer 3

1. 3. There are no notable methodological contributions. The factors were not allowed any dynamicity, which is a problem given that gene expression changes significantly during the profiled time course. The RMSE values of NMF were small, but that is likely an artifact of scaling all gene expressions between 0 and 1.

The NMF analysis is explicitly aimed at capturing the temporal expression patterns which, as the reviewer rightly points out, are highly dynamic. In NMF, the temporal expression of gene g at timepoint t, X_{gt}, is represented as a weighted sum of the expression of metagenes/behaviors, H_{mt},

$$X_{gt} = \sum_m W_{gm} H_{mt},$$

where m is the metagene and t is the timepoint. This appears as equation 1 in the manuscript. Dropping g and writing X and H_m as functions of time, the factorization for a single gene may be rewritten as

$$X(t) = \sum_m W_m H_m(t).$$

The factorization therefore accounts for the temporal dynamics of each gene via the time-dependence of the $H(t)$ factor, i.e. the expression of the metagene (behavior). We realize that this may not have

been clear and have added an graphical example of how a gene's temporal dynamics is represented as a weighted sum of the behavioral/metagene temporal dynamics in Figure 2.

If by this comment the reviewer meant the introduction of time-dependent weights W_{gm} in addition to the time dependence of the behaviors H_{mt}, it is unnecessary and probably ill-advised for two reasons. Time-dependent weights are unnecessary because more dynamicity may be introduced simply by increasing the number of metagenes/behaviors M to achieve arbitrarily low RMS, achieving a perfect fit in the limit when the number of behaviors equals the number of time-points ($M = T$). This is because one can choose $\mathbf{W} = \mathbf{X}$ and $\mathbf{H} = \mathbf{I}$, where $\mathbf{I}$ is the identity matrix, when $\mathbf{H}$ is an $M \times M$ matrix. Doing so is ill-advised because it is over-fitting and no dimensionality reduction is achieved. Instead, we chose the number of behaviors by progressively increasing their number until no new unique dynamical expression patterns appeared.

2. 4. Follow-up of point #3, it is important to test the assumption that the non-negative factors need not be dynamic. Does the presented NMF approach work well on single-cell lineage tracing data?

We thank the reviewer for this suggestion and have tested the NMF algorithm on Tusi *et al.*'s bone marrow data. It works well with non-varying weights/factors; the inferred behaviors correspond to the major lineages as well as intermediate developmental stages. We performed NMF on the single-cell data both with the maximum expression of each gene scaled to 1 (Fig. S18 and line numbers 445–463) and without (Fig. S19). NMF on scaled data infers behaviors corresponding to the erythroid (behavior 14), neutrophil (behavior 2), monocyte (behavior 17), lymphoid (behavior 11), and basophil (behavior 5) lineages as well as intermediate states such as early erythrocyte (behavior 9), early neutrophil (behavior 13), granulocyte-monocyte (behavior 12), megakaryocyte-erythrocyte (behavior 15) etc. It is worth noting that these behaviors match Tusi *et al.*'s marker-based lineage assignments even though they were inferred by NMF in an unsupervised manner without any foreknowledge of lineages or developmental stages. Just as with NMF on the PUER time-series data, the behaviors H vary from cell to cell but their weights W remain constant. This demonstrates the sufficiency of constant weights to recapitulate genome-wide gene expression patterns in two independent datasets.

In contrast, NMF on unscaled single-cell data (Fig. S19) infers behaviors corresponding to only three lineages: late erythrocyte, late neutrophil, and progenitors. This is because a few genes with very high absolute expression in these lineages dominate the factorization process. A good approximation may be achieved by reproducing the expression of only a few highly expressed genes without needing to recapitulate the expression of the vast majority of the genes. This supports our choice to scale the maximum expression of each gene to 1 prior to performing NMF.

3. 1. The authors have dedicated major effort to justify that their choice of PUER cells reflect in vivo biology. However, the conclusions that PUER cells approach BM cells and PUER neutrophils and macrophages are closer in their transcriptomic state to neutrophils and macrophages developing in vivo, do not convincingly justify that using PUER cells can reveal the intermediate cell states and dynamic mechanisms of hematopoiesis. So, the mechanistic value of the conclusions is questionable.

We would like to respectfully disagree with the idea that perturbed states do not inform our mechanistic understanding of endogenous hematopoeisis. First of all, hematopoiesis exhibits a lot of cell-type plasticity and *in vitro* transdifferentiation and reprogramming experiments have a long and fruitful history in the field (1, 2). Second, there is also a long history of using inducible cell lines, such as

PUER (3, 4, 5), G1ME (6), FDCP (7, 8, 9, 10) and others to gain a mechanistic understanding of endogenous hematopoiesis. These inducible systems are accepted as models of hematopoiesis and many commonly accepted hematopoietic GRNs were inferred from analyses of such perturbed systems (5, 10). In fact, the very insight that PUER differentiation is akin to reprogramming (Fig. 1), which could not have been arrived at without the analysis we present here, provides a new perspective on these earlier classic studies.

While the intermediate states PUER cells transit are not going to be exactly the same as those traversed by cells *in vivo*—something that is true of any other model system, e.g. ESC or iPSC differentiation—more general inferences are nevertheless relevant and informative. The differentiation is driven by the expression of PU.1, an important player *in vivo*; the TFs and genomic regulatory sequences are exactly the same in PUER cells and endogenous cells so the GRNs involved are also going to be the same. Our main conclusions about sharp transitions or cascading waves do not depend strongly on the specific states traversed. That these conclusions might be general is supported by the observation of sharp transitions in B-cell-to-Macrophage transdifferentiation in a completely independent system (11). With careful and nuanced interpretation, our results should provide insight into endogenous hematopoiesis and reprogramming experiments in addition to imparting a new perspective to past studies utilizing inducible systems.

4. 2. As a follow-up of point #1, we should avoid accepting batch-effect-corrected results at their face value. Newer methods can identify what information is lost due to batch-effect corrections. The authors should consider those methods.

The reviewer is concerned that information loss during batch correction could confound downstream analyses such as clustering or differential expression analysis (12, 13). Although this is an important problem in conventional scRNA-Seq analyses, it does not affect our results since we do not use the batch-corrected PUER data for any downstream analysis. Our main goal in batch correction was to identify which *in vivo* bone-marrow cell types differentiating PUER cells most resemble in their transcriptomic state in order to properly situate PUER differentiation in relation to the hematopoietic hierarchy. Excepting this limited use of batch-corrected values, only uncorrected PUER data were used in all subsequent analyses such as NMF, DEG, or GO. It is also important to point out that the Tusi *et al.* data remained unmodified by virtue of being the reference dataset to which the PUER data were registered.

Finally, even though we did not use the corrected PUER data, we have checked whether MNNCorrect caused sufficient information loss to alter the transcriptomic states of different PUER samples relative to their known identities. The figure below plots the top two principal components of the PUER expression data before and after correction, annotated by condition and timepoint. Except for very minor shifts, the relative placement of the samples by replicate, condition, and timepoint is preserved after correction, showing that no significant information loss has occurred.

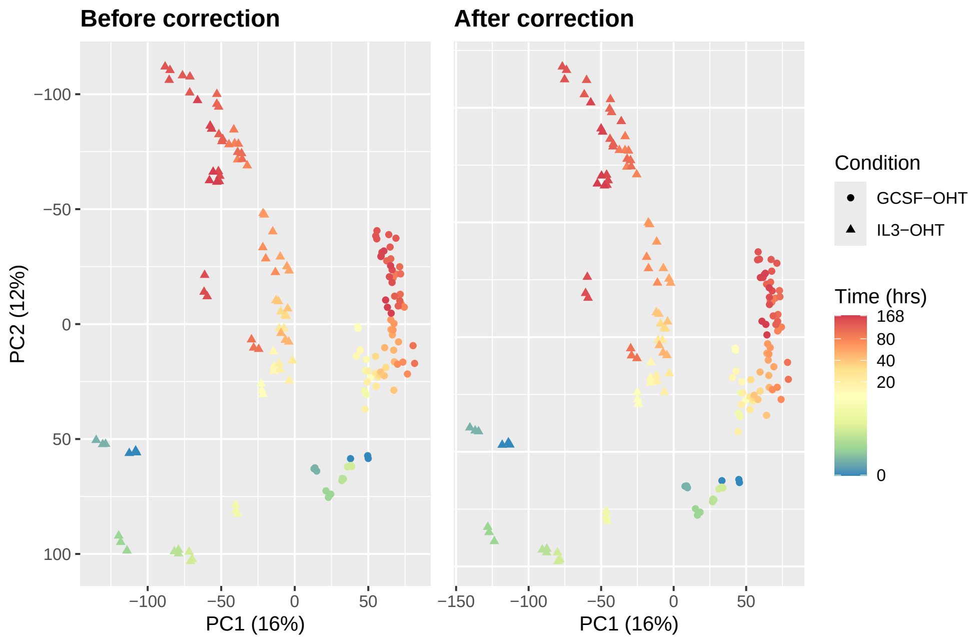

5. 5. The predictive value of this work is questionable: there were opportunities to derive predictive ODE models, which could help testing on held-out time points.

We agree with the reviewer that deriving predictive ODE models is an important goal. However we regard it as being out of the scope of the present work, which is focused on characterizing the dynamical process by which differences in initial conditions are transduced into genome-wide transcriptomic differences through experiment and data analysis. We hope the reviewers and editor will agree that ODE modeling is better suited to future follow-up studies.

6. 6. It is also unclear if/how the conclusions fit with prior single-cell studies of cell-fate tracing during hematopoiesis (e.g., Weinreb et al.'s data).

We have addressed this comment and similar comments by reviewer 1 by providing detailed comparisons of the expression of individual marker/TF genes and GRNs between our and Tusi *et al.*'s datasets (Fig. S6-S11). The single-cell bone marrow data are static snapshots and do not contain explicit temporal information. Our comparisons therefore are limited to conclusions about the order of gene expression changes. There is a great deal of congruence between the granulocyte-monocyte marker/TF expression patterns and GRNs inferred from our data and those inferred from single-cell data.

Other points

Reviewer 1

1. Fig. 1D: it is hard to visualize the in vitro differentiated cells because they are very sparse compared to the cells from bone- marrow.

We have increased the point size of PUER cells.

2. Fig. 2C; labeling of the earlier time points on the heatmap will be useful as a lot of the discussion focuses on early time points (8 hrs)

We have done so.

3. Fig. 3A-B: It would be useful to label the specific genes that are turned on or off that corresponding to known gene regulatory events in granulocyte or macrophage differentiation.

We have done so.

4. Fig. 3E-F: These Venn diagrams are hard to interpret and my opinion do not clearly convey the author's points.

We have explained the Venn diagrams better in the text.

5. Fig. 5: The 3D heatmap is hard to visualize. It would be more useful to do a 2D heatmap that is annotated and also unfolds over time, see for instance ( )

The goal of the 3D heatmaps is to illustrate non-negative matrix factorization so that one can associate genes' expression patterns with the behaviors they have high weight for, something this is not possible with a 2D heatmap. However, we appreciate the reviewer's point and have now included a 2D heatmap organized by dominant behavior (Fig. S16) that has the same patterns as Figure 5.

6. Figure legends (Fig. 2 and thereafter): It would be useful the experiment from which the data is derived, even if briefly (e.g. one sentence indicating that the data were derived from the experiment as performed in Fig. 1). It would also be useful to briefly describe the computational method used (e.g. NMF for Fig. 4)

We have done so.

Reviewer 2

1. 1. The methods state 4 biological replicates were collected for each timepoint (line 625) but many of the figures show only 3 replicates (e.g. Fig S1). Presumably the methods is a typo, or one replicate from each timepoint was held back?

RNA was extracted from only 3 replicates, the fourth one being a backup. We have clarified in the Methods section.

2. 2. I assume from the methods that 4OHT induction of the GCSF pre-treated samples is in the continued presence of GCSF but it would be helpful to add this to the text.

We have stated this explicitly in the Methods section.

3. 3. In fig S1, although the outlier timepoints that are identified in the text (96h and 144h) can be seen in the clustering, it would be helpful to highlight them in the timeline.

We have annotated the outliers explicitly in Figure S1.

4. 4. Figures S11 and S12 show the GO term "negative regulation of cell cycle" is enriched in both Behaviors 1 and 2, although Behavior 2 also shows enrichment of positive regulation of cell cycle. Also, the genes in Behavior 2 are transiently downregulated after addition

of 4OHT but then significantly upregulated throughout most of the timecourse. I am not sure why this leads the authors to suggest that GCSF pre-treatment and addition of OHT in the presence of GCSF have opposing effects on the cell cycle (line 480), and they should explain this more clearly, or remove it from the text.

We have limited our argument to behavior 1, on which GCSF and OHT have unambiguously opposing effects. The genes exhibiting behavior 1 tend to be upregulated by GCSF treatment and downregulated by OHT treatment. The top genes for this behavior tend to involve the cell cycle, evinced by the enrichment of cell cycle GO terms. Therefore, GCSF and OHT have opposing effects on cell cycle genes.

We would also point out that there are many other enriched terms that are directly or indirectly related to cell cycle terms besides "negative regulation of cell cycle", such as "chromosome segregation", "DNA replication" etc.

References

1. Graf T, Enver T. Forcing cells to change lineages. Nature. 2009;462(7273):587–94. doi:10.1038/nature08533.

2. Look AT. Oncogenic transcription factors in the human acute leukemias. Science. 1997;278(5340):1059–64.

3. Walsh JC, DeKoter RP, Lee HJ, Smith ED, Lancki DW, Gurish MF, et al. Cooperative and antagonistic interplay between PU.1 and GATA-2 in the specification of myeloid cell fates. Immunity. 2002;17(5):665–76.

4. Dahl R, Walsh JC, Lancki D, Laslo P, Iyer SR, Singh H, et al. Regulation of macrophage and neutrophil cell fates by the PU.1:C/EBPalpha ratio and granulocyte colony-stimulating factor. Nat Immunol. 2003;4(10):1029–36. doi:10.1038/ni973.

5. Laslo P, Spooner CJ, Warmflash A, Lancki DW, Lee HJ, Sciammas R, et al. Multilineage transcriptional priming and determination of alternate hematopoietic cell fates. Cell. 2006;126(4):755–66. doi:10.1016/j.cell.2006.06.052.

6. Stachura DL, Chou ST, Weiss MJ. Early block to erythromegakaryocytic development conferred by loss of transcription factor GATA-1. Blood. 2006;107(1):87–97. doi:10.1182/blood-2005-07-2740.

7. Ford AM, Healy LE, Bennett CA, Navarro E, Spooncer E, Greaves MF. Multilineage phenotypes of interleukin-3-dependent progenitor cells. Blood. 1992;79(8):1962–71.

8. Heyworth CM, Alauldin M, Cross MA, Fairbairn LJ, Dexter TM, Whetton AD. Erythroid development of the FDCP-Mix A4 multipotent cell line is governed by the relative concentrations of erythropoietin and interleukin 3. Br J Haematol. 1995;91(1):15–22.

9. Huang S, Guo YP, May G, Enver T. Bifurcation dynamics in lineage-commitment in bipotent progenitor cells. Developmental Biology. 2007;305(2):695–713. doi:https://doi.org/10.1016/j.ydbio.2007.02.036.

10. May G, Soneji S, Tipping AJ, Teles J, McGowan SJ, Wu M, et al. Dynamic analysis of gene expression and genome-wide transcription factor binding during lineage specification of multipotent progenitors. Cell Stem Cell. 2013;13(6):754–68. doi:10.1016/j.stem.2013.09.003.

11. Choi J, Lysakovskaia K, Stik G, Demel C, Soding J, Tian TV, et al. Evidence for additive and synergistic action of mammalian enhancers during cell fate determination. eLife. 2021;10:e65381. doi:10.7554/eLife.65381.

12. Emmanúel Antonsson S, Melsted P. Batch correction methods used in single cell RNA-sequencing analyses are often poorly calibrated. bioRxiv. 2024; p. 2024.03.19.585562. doi:10.1101/2024.03.19.585562.

13. Chazarra-Gil R, van Dongen S, Kiselev VY, Hemberg M. Flexible comparison of batch correction methods for single-cell RNA-seq using BatchBench. Nucleic Acids Res. 2021;49(7):e42. doi:10.1093/nar/gkab004.
