# Supplementary material for: The differentiation of myeloid progenitors is effected by cascading waves of coordinated gene expression that remodel cellular physiology in a characteristic sequence
Source: PLoS Comput Biol. 2026 May 12;22(5):e1014276. doi: 10.1371/journal.pcbi.1014276 (PMC13189416; doi:10.1371/journal.pcbi.1014276)
Supplement: Attachment — Submitted filename: puerdiff_rev1_response_to_reviewers.pdf [file pcbi.1014276.s041.pdf]

Responses to reviewer comments on manuscript #PCOMPBIOL-D-25-01103R1

Overview: We thank the editor and reviewers for their insightful critiques. We have revised the manuscript accordingly.

A point by point description of the changes follows. *Note:* New supplementary figures added and the line numbers of revised text are highlighted in red below.

Major points

Reviewer 2

1. The reviewer points out that the results of the switch experiment do not rule out the possibility that a majority of the cells could have undergone irreversible commitment to the neutrophil lineage and that the population retaining monocyte potential may be a very small fraction of the total. In this scenario, it would not be OK to claim that PUER cells model a bipotential fate decision.

   The reviewer suggests that this possibility could be checked by considering the numbers or viabilities of the cells in the switch experiment. If only a small fraction of cells retain monocyte potential after GCSF pre-treatment, the majority being irreversibly committed to the neutrophil fate, then the latter population must die in order to produce a monocyte dominated cell-type distribution at 96h after the switch. Therefore, low viability or cell numbers at 96h in the switch experiment would support the view that only a small number of cells are bipotential after GCSF treatment. Viability comparable to the IL3 condition would suggest instead that no die-off had occurred and that a significant fraction of the population remained bipotential after GCSF pre-treatment.

   > Point 1 – re whether G-CSF pre-treated cells remain bi-potential and therefore the activation of PU-ER by OHT triggers neutrophil commitment of a bi-potential cell (as distinct from modelling the earliest stages of neutrophil differentiation, post-commitment). The authors have performed the suggested "switch" experiment, and I am satisfied that this demonstrates that after pre-treatment with G-CSF, at least some of the cells remain able to respond to IL-3 and are therefore not irreversibly committed to neutrophil differentiation. However, as Fig S12 presents the 96h data in the form of cell percentages, it remains a formal possibility that the monocytic cells observed after 96h in the switch condition could originate from only a small proportion of the cells pre-treated with G-CSF (which have retained bi- lineage potential), with the majority of the cells in fact having undergone some degree of neutrophil commitment. Any such neutrophil-committed cells would presumably be present in the RNA samples harvested at the time of OHT addition in the G-CSF time-course. This possibility can be readily excluded by the authors simply confirming in the text or the figure legend that the cell numbers were similar throughout the IL-3 and switch experiments. If the authors did not record cell numbers (or % viability?) in the switching experiment and so are unable to exclude this possible caveat, they should consider softening some of their statements. For example, to reconsider the statement (line

220) that “the effects of G-CSF pre-treatment are completely reversible. . . .” . It seems a little unlikely that the large effect of G-CSF pre- treatment on gene expression (Fig 2A; -48h vs 0h) does not include an impact on some genes involved in lineage choice. I do not think it is particularly problematic if it remains somewhat open to debate whether or not the gene expression analysis of G-CSF differentiation captures the moment of lineage commitment, but is important that this is As an additional point, fig S12E has error bars but the legend does not indicate the number of replicate experiments clarified. performed.

The distributions shown in Fig. S12 were generated by scoring a constant number of cells so that the conditions could be compared. However, the flow cytometry data (Fig. S13) included a LIVE/DEAD stain so we have accurate assessments of viability. The viability of the cells in the switch experiment was high (93%) and comparable to that in IL3 conditions (93%). This supports the interpretation that a significant proportion of the cells remain bipotential after GCSF pre-treatment. The viability numbers are mentioned and this possibility is discussed in the results.

However, we agree with the reviewer that it is not possible to conclusively establish the bipotential nature of the cells without single-cell lineage tracing. Therefore, we have also softened the claims as suggested by the reviewer.

We have included the replicate numbers that Fig. S12E is based upon in the legend.

Line numbers 219–230 and 1134–1136.

2. Fig S20 : As the authors say, it is clear that the gene set that is upregulated in the first hour of differentiation is enriched for genes they identify as bound by PU1 at 48h (either close to the TSS or more distally), and it is reasonable to assume that much if not all of this upregulation is due to rapid PU1 binding after its activation by OHT. I am surprised that this figure only contains the DEGs from 0h vs 1h to represent early changes in gene expression, and wonder if I am missing something? In Fig 6A, it is apparent that in the IL3 condition, relatively few DEGs are detected in 0h vs 1h, compared with 1h vs 2h, 2h vs 3h, 4h vs 8h, and even 8h vs 12h. Given this, the use in Fig S20 of only the 0h vs 1h DEGs from within the first 12 hours of differentiation seems odd, especially as (i) the 0h vs 1h DEGs have not been included in Figs 6B and 6C, and (ii) the authors have identified that a key transition in gene expression occurs at 8 hours. The most interesting insights might come from those genes that are differential between 8h and either 4h or 12h (or both). Since the authors already have the GE and the PU1 binding data, one or more of the other early timepoint comparisons should be added to Fig S20.

We thank the reviewer for catching this oversight on our part. We have added all consecutive timepoint comparisons up to 12h to Fig. S20. PU.1 binding is enriched near all early DEGs except to the proximal regions of DEGs between 1h and 2h and 3h and 4h, strengthening the inference that PU.1 binding drives early changes in IL3 conditions. One of the exceptions, 3h vs. 4h, behaves erratically due to having very few DEGs (Fig. 6A). We have also removed the enrichment of PU.1 binding near genes differentially expressed in GCSF since, as the reviewer pointed out, inferences about GCSF are not possible without PU.1 binding data from GCSF.

Line numbers 505–517.

3. Fig S21 : Here the authors look for enrichment of PU1-bound genes (defined as genes/loci bound by PU1/ER after 48h of culture in IL3 + OHT) within the 10 patterns of gene

expression(Behaviors 1 – 10) defined in Figure 4. This approach gives greater insight into the likely role of PU.1 in initiating and supporting differentiation, as it makes more use of the dynamics of gene expression across the 168 hours. The most marked enrichments of PUER binding are shown to be within 1 kb of the TSS of genes with expression Behaviors 2,3, 4 and 10. In the description of the results, the authors need to take more account of the fact that the binding data is derived from a "snapshot" of cells differentiated for 48 hours in IL-3, and acknowledge that it may not be correct to infer that PU1 binding around a given set of genes would be the same in neutrophil conditions as in IL3. For example, they use the enrichment of binding to genes with Behaviors 4 and 10 to support the statement (line 523) that "PU1 tends to regulate genes... . . . ...turned on permanently at the end". For Behavior 10 genes, this is a reasonable interpretation, as they are upregulated after 60h in IL-3, and enrichment of PU1 binding is seen at 48h in IL-3. The lack of upregulation of these genes in G-CSF could be attributed to a lack of PU.1 binding or to other regulatory factors. But the situation for Behavior 4 genes is more complicated, as they are not upregulated in IL-3 – even though this is where the increase in PU1 binding was seen – but are upregulated in G-CSF, where PU1 binding can only be inferred. Inferring the contribution of PU1 binding to changes in gene expression is not a problem, as long as the authors address more specifically how strongly each inference they draw is supported by the experimental data.

We thank the reviewer and agree that it is not possible to reach conclusions about behaviors expressed exclusively in GCSF based on PU.1 binding data from IL3 conditions. We have narrowed the scope of the conclusions to behaviors having expression in IL3.

Line numbers 524–530.

Reviewer 3

Our point about "The factors were not allowed any dynamicity" was probably unclear to the authors. We meant that the H_t factors are not functions of time (i.e., H(t)) by design. The H_t factors are fit independently for each timepoint, there is no conditional dependency of H_t+1 on H_t. This should be clarified in their Methods and discussed as future works.

We have clarified in the Methods section that no conditional dependence has been imposed upon the time evolution of $H_m(t)$ in our implementation of NMF. We also mention in the Discussion section that requiring the temporal behaviors to satisfy such constraints could be used to test specific hypotheses about temporal behaviors in the future.

Line numbers 791–795 and 990–995.
